# Atom-specific persistent homology and its application to protein flexibility analysis

**DOI:** 10.1515/cmb-2020-0001

**Published:** 2020-02-17

**Authors:** David Bramer, Guo-Wei Wei

**Affiliations:** Department of Mathematics, Michigan State University, MI 48824, USA; Department of Mathematics, Michigan State University, MI 48824, USA; Department of Biochemistry and Molecular Biology, Michigan State University, MI 48824, USA; Department of Electrical and Computer Engineering, Michigan State University, MI 48824, USA

**Keywords:** Atom-specific topology, Element-specific persistent homology, Protein flexibility, Gradient boosting tree, Convolutional neural network

## Abstract

Recently, persistent homology has had tremendous success in biomolecular data analysis. It works by examining the topological relationship or connectivity of a group of atoms in a molecule at a variety of scales, then rendering a family of topological representations of the molecule. However, persistent homology is rarely employed for the analysis of atomic properties, such as biomolecular flexibility analysis or B-factor prediction. This work introduces atom-specific persistent homology to provide a local atomic level representation of a molecule via a global topological tool. This is achieved through the construction of a pair of conjugated sets of atoms and corresponding conjugated simplicial complexes, as well as conjugated topological spaces. The difference between the topological invariants of the pair of conjugated sets is measured by Bottleneck and Wasserstein metrics and leads to an atom-specific topological representation of individual atomic properties in a molecule. Atom-specific topological features are integrated with various machine learning algorithms, including gradient boosting trees and convolutional neural network for protein thermal fluctuation analysis and B-factor prediction. Extensive numerical results indicate the proposed method provides a powerful topological tool for analyzing and predicting localized information in complex macromolecules.

## 1 Introduction

In recent years tools from topology have been successfully applied to protein analysis [1, 2, 3, 4, 5, 6]. Topology offers one of highest level of abstractions of geometric data and allows one to infer high dimensional structure from low dimensional topological invariants. However, conventional topology oversimplifies geometry and thus lacks descriptive power for most real world problems. Persistent homology (PH) overcomes this difficulty by introducing a filtration parameter that describes the geometry in terms of a family of Betti numbers at various scales known as a barcode [7, 8, 9, 10]. Indeed, three dimensional (3D) protein spatial information from a protein data bank (PDB) file can be converted into a family of simplicial complexes. One can apply tools from algebraic topology to convert structural information into global topological invariants that provide a useful representation of biomolecular properties [11]. However, for quantitative biomolecular analysis and prediction, persistent homology alone neglects chemical and biology information. Element-specific persistent homology has been introduced to incorporate chemical and biological information into topological invariants [12, 13]. Similarity and differences between barcodes from different molecules can be measured by Wasserstein [14] and/or Bottleneck [15] distances. However, the previous applications of persistent homology and element-specific persistent homology are for the modeling and prediction of molecule-level thermodynamical or structural properties, such as protein-ligand binding affinities [13], protein folding free energy changes upon mutations [12, 16], drug toxicity [17], solubility, partition coefficient [18], and drug virtual screening (ligand and decoy classification) [19]. Essentially, topology is a global tool that examines the connectivity and relationship among many atoms in a neighborhood as a whole. High dimensional topological invariants, such as Betti 1 and Betti 2, describe the collective behavior of many atoms [20]. Therefore, it is not clear how to represent atomic level property, such as the B-factor of an atom, by persistent homology.

In proteins, beta factor (B-factor) or (Debye-Waller factor is a measure of the attenuation of X-ray scattering caused by thermal motion. The amplitude of the thermal motion of an atom is theoretically proportional to its B-factor during the structure determination from X-ray diffraction data. It is well known that biomolecular flexibility provides an important link between its structure and function. In particular, it has been shown that intrinsic structural flexibility correlates to meaningful protein conformational variations, reactivity and enzymatic function [21]. As such, the accurate prediction of protein B-factor is essential to our understanding of protein structure, function and dynamics [22].

Early methods used to predict protein B-factor were derived from Hooke’s Law and are known as elastic mass-and-spring networks. In these models, alpha carbons (C_*α*_) of biological macromolecules are treated as a mass and spring network and motions are predicted based on a harmonic potential. Given a protein, each C_*α*_ is represented as a node in the network and edges are weighted based on a potential function. Nodes are connected by an edge if they fall within a pre-defined euclidean cutoff distance. This captures the local covalent and non-covalent interactions between an individual atom and nearby atoms. One of the first mass- and-spring methods used for protein B-factor prediction is normal mode analysis (NMA). Like most B-factor prediction methods, NMA is independent of time and uses a Hamiltonian interaction matrix. Eigenvalues of the matrix system correspond to characteristic frequencies of the protein and these frequencies correlate with protein B-factors. Low-frequency modes correlate with cooperative motion and can be useful for hinge detection and domain motion. NMA has also been successfully implemented to understand the deformation of supramolecular complexes. [21, 23, 24, 25]

Elastic network model (ENM) was introduced as a more efficient model that significantly reduces computational cost compared to NMA through the use of a simplified spring network [26]. A specific example is anisotropic network model (ANM) [27]. Gaussian network model (GNM) further reduces the computational cost by ignoring the anisotropic motion, rendering a more accurate method for protein C_*α*_ B-factor analysis [28, 29, 30].

All of the aforementioned methods depend on matrix diagonalization, which has the computational complexity of $O(N^{3})$, where *N* is the number of matrix atoms involved in the analysis. Recently, flexibility and Rigidity Index (FRI) methods have been proposed as a geometric graph approach to further reduce the computational cost. FRI methods rely on constructing a distance matrix using radial basis functions to scale atom to atom distance non-linearly [31]. All versions of FRI produce a flexibility index, that correlates to the B-factor, for each C_*α*_. Several versions of FRI have been developed. Among them, fast FRI (fFRI) is of O(N) in computational complexity [32]. FRI methods are also more accurate than all of the earlier algebraic graph-based methods. Additionally, anisotropic FRI (aFRI) provides high quality anisotropic motion analysis [32]. Moreover, using several radial basis functions with different parametrizations, the multiscale flexibility rigidity index (mFRI) can successfully capture multiscale atomic interactions [33].

More recently, the authors introduced a multiscale weighted colored graph (MWCG) model. The MWCG is another geometric graph theory model that has been shown to be the best B-factor prediction model to date. First, element-specific interaction subgraphs are constructed based on selected atomic interactions between certain element types. Atoms are represented as graph nodes and subgraphs are generated using pairs of atoms of certain elements (e. g., carbon, nitrogen, oxygen, sulfur). A centrality metric that uses radial basis functions is applied to pairwise interactions in each subgraph. By varying the parametrization of the radial basis functions the MWCG model can capture multiple protein interaction scales. MWCG is unique in its ability to utilize both element specific and multiscale interactions for improved B-factor prediction [34]. Most recently, MWCG is incorporated with machine learning algorithms for across-protein blind predictions of protein B-factors [35].

The objective of the present work is to extend the utility of persistent homology for atomic level property modeling and prediction. To this end, we introduce atom-specific persistent homology (ASPH) to create a local atomic representation of an atom using a global topological tool in a novel way. Specifically, ASPH constructs a pair of conjugated sets of point clouds or atoms centered around the atom of interest. The first set of a pair of conjugated sets of atoms for a given atom is selected by a local sphere of radius *r_c_* around the atom of interest. The second set of atoms is defined by excluding the atom of interest in the first set. Conjugated simplicial complexes, conjugated chain groups, conjugated homology groups as well as conjugated persistence barcodes or diagrams are induced by an identical filtration. Conjugated persistence barcodes are compared with Bottleneck and Wasserstein metrics. The resulting distance provides a global topological representation of the localized atomic property, such as protein flexibility analysis and atomic-level protein B-factor information. Obviously, the proposed atom-specific topology can be applied to a wide variety of chemical and biological problems where atomic properties are measured, such as the chemical shifts of nuclear magnetic resonance (NMR), the B-factors of X-ray structure determination, and the shift and line broadening of other atomic spectroscopy.

We focus on protein C_*α*_ B-factor prediction but the approach provided in this work is a general framework that can be used to predict B-factors of any atom in a protein. First, we use the generated atom-specific persistent homology features to fit B-factors within a given protein using linear least squares minimization. Note that this method does work for blind B-factor predictions across proteins. Additionally, the atom-specific persistent homology features are combined with other local and global protein features to construct machine learning models for the blind prediction of protein B-factors across different proteins. Moreover, image-like multiscale atom-specific persistent homology features are generated using an early technique [36]. These image like features, together with other features, are fed into convolutional neural networks (CNN). Training and validation are carried out using a large and diverse set of proteins from the protein data bank (PDB). We demonstrate that the proposed method offers some of the best results for blind B-factor predictions of a set of 364 proteins.

## 2 Methods and algorithms

### 2.1 Atom-specific persistent homology

#### 2.1.1 Overview

Topology describes (continuous) objects in terms of topological invariants, i.e., Betti numbers. Betti-0, Betti-1, and Betti-2 which can be interpreted as connected components, rings, cavities, etc. Table 1 provides examples of the Betti numbers of a point, circle, sphere, and torus.

**Figure 1:**
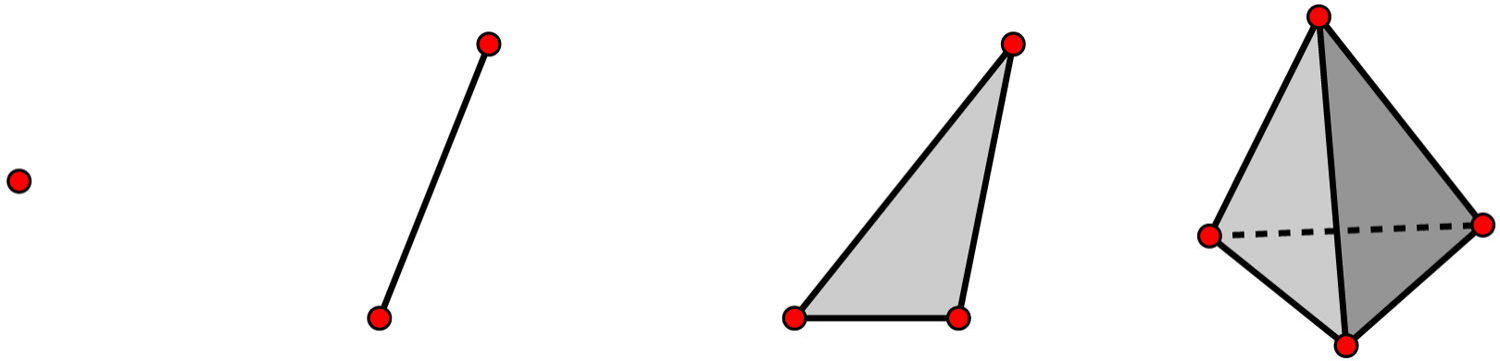
From left to right an example of a 0-simplex, 1-simplex, 2-simplex, and 3-simplex.

Given discrete data points, such as a point cloud or the set of atoms in a molecule, we use simplicial complexes to describe the topological relationship, or connectivity of the point cloud, to systematically identify topological invariants. First, a few simplicial complexes, as shown in Figure 1, are made up of vertices, edges, triangles, and tetrahedrons, denoted 0-simplex, 1-simplex, 2-simplex, and 3-simplex, respectively. Homology groups constructed from simplicial complexes give rise topological invariants. Given discrete dataset or a set of protein atoms, nontrivial topological information is generated by persistent homology. This introduces a filtration parameter to create a family of simplexes, which leads to a family of simplicial complexes, homology groups and associated topological invariants. By continuously varying the filtration parameter over an interval, the topological relationship among a given set of atoms is systematically reset, rendering a family of homology groups and corresponding topological invariants, which can be plotted as a persistence diagram, or a set of barcodes. Both persistence diagrams and barcodes record the birth and death (appearance and cessation) of Betti numbers during the filtration process. Many simplicial complex definitions, which determine the rules of the corresponding topological relationship, have been proposed. Specifically, Vietoris-Rips (VR) complex, Čech complex, and alpha complex are commonly used.

Persistent homology allows the extraction of topological invariants that are embedded in the high dimensional data space of biomolecules. The resulting topological invariants over the filtration, i.e., persistence diagrams or persistence barcodes of different molecules can be compared using Bottleneck and Wasserstein distances.

The goal of atom-specific persistent homology is to extract topological information of a given atom in a molecule. To embed local atomic information into a global topological description, we construct a pair of conjugated sets of point clouds, namely the original dataset and a datset excluding the atom of interest. The Bottleneck and Wasserstein distances between these two persistence diagrams reveal the desirable topological information of the given atom.

#### 2.1.2 Simplex and simplicial complex

A (geometric) simplex is a generalization of a triangle or tetrahedron to arbitrary dimensions. A *k*-simplex is a convex hull of *k* + 1 vertices represented by a set of affinely independent points

(1) $$\sigma =\{\lambda_{0}u_{0}+\lambda_{1}u_{1}+\ldots +\lambda_{k}u_{k}|\sum \lambda_{i}=1,\lambda_{i}\geq 0,i=0,1,\ldots ,k\},$$

where $\{u_{0},u_{1},\ldots ,u_{k}\}\subset R^{d}$ with *d* ≥ *k* is the set of points, *σ* is the *k*-simplex, and constraints on *λ_i_*,’s ensure the formation of a convex hull. An affinely independent combination of points can have at most *k* + 1 points in $R^{k}$. For example a 1-simplex is a line segment, a 2-simplex a triangle, and a 3-simplex a tetrahedron. A subset of the *k* + 1 vertices of a *k* simplex with *m* + 1 vertices forms a convex hull in a lower dimension and is called an *m*-face of the *k*-simplex. An *m*-face is proper is *m* < *k*. The boundary of a *k*-simplex *σ*, is defined as the alternating sum of its (*k* + 1) faces, given as

(2) $$\partial_{k}\sigma =\sum_{i=0}^{k}(-1{)}^{i}[u_{0},\ldots ,{\hat{u}}_{i},\ldots ,u_{k}],$$

where [$u_{0},\ldots ,{\hat{u}}_{i},\ldots ,u_{k}$] denotes the convex hull formed by vertices of *σ* with the vertex *u_i_* being excluded and ∂_*k*_ is called the boundary operator. A collection of finitely many simplicies forms a simplicial complex denoted by K. All simplicial complexes satisfy the following conditions.

1. Faces of any simplex in K are also simplices in K.
2. The intersection of any two simplicies *σ*_1_, $\sigma_{2}\in K$ is a face of both *σ*_1_ and *σ*_2_.

#### 2.1.3 Homology

Given a simplicial complex K, a *k*-chain *c_k_* of K is a formal sum of the *k*-simplices in K and is defined as *c_k_* = ∑ *a_i_σ_i_* where *σ_i_* are the *k*-simplices and *a_i_*’s coefficients. Generally, *a_i_* are element of a field such as R, Q, or $Z_{n}$. Computationally, it is common to choose *a_i_* to be in $Z_{2}$. The group of *k*-chains in K, denoted *C_k_*, forms an Abelian group under addition in modulo two. This allows us to extend the definition of the boundary operator introduced in Eq. (2) to chains.

The boundary operator applied to a *k*-chain *c_k_* is defined as

(3) $$\partial_{k}c_{k}=\sum a_{i}\partial_{k}\sigma_{i},$$

where *σ_i_*’s are *k*-simplices. The boundary operator is a map from $C_{k}$ to $C_{k-1}$, which is also known as a boundary map for chains. Note that in $Z_{2}$, the boundary operator ∂_*k*_ satisfies the property that ∂_*k*_ ‘ ∂_*k*+1_ *σ* = 0 for any (*k* + 1)-simplex *σ* following the fact that any (*k* – 1)-face of *σ* is contained in exactly two *k*-faces of *σ*. The chain complex is defined as a sequence of chains connected by boundary maps with decreasing dimension and is denoted

(4) $$\ldots \to C_{n}(K)\overset{\partial_{n}}{\to }C_{n-1}(K)\overset{\partial_{n-1}}{\to }\ldots \overset{\partial_{1}}{\to }C_{0}(K)\overset{\partial_{0}}{\to }0.$$

The *k*-cycle group and *k*-boundary group are then defined as kernel and image of ∂_*k*_ and ∂_*k*+1_ respectively, and

(5) $$Z_{k}=\text{Ker}\partial_{k}=\{c\in C_{k}\;|\;\partial_{k}c=0\},$$

(6) $$B_{k}=\text{Im}\partial_{k+1}=\{c\in C_{k}\;|\;\exists d\in C_{k+1}\;:\;c=\partial_{k+1}d\},$$

where $Z_{k}$ is the *k*-cycle group and $B_{k}$ is the *k*-boundary group. Since ∂_*k*_ ‘ ∂_*k*+1_ = 0, we have $B_{k}\subseteq Z_{k}\subseteq C_{k}$. Then the *k*-homology group is defined to be the quotient group of the *k*-cycle group modulo the *k*-boundary group,

(7) $$H_{k}=Z_{k}∕B_{k}$$

where $H_{k}$ is the *k*-homology group. The *k*th Betti number is defined to be rank of the *k*-homology group as $\beta_{k}=\text{rank}(H_{k})$.

#### 2.1.4 Filtration and persistence

For a simplicial complex K, we define a filtration of K as a nested sequence of subcomplexes of K,

(8) $$\emptyset \subseteq K_{0}\subseteq K_{1}\ldots \subseteq K_{n}=K$$

In persistent homology, the nested sequence of subcomplexes usually depends on a filtration parameter. The persistence of a topological feature is denoted graphically by its life span with respect to filtration parameter. Subcomplexes corresponding to various filtration parameters offer the topological fingerprints over multiple scales. The *k^th^* persistence Betti number $\beta_{k}^{i,j}$ is given by the ranks of the *k^th^* homology groups of $K_{i}$ that are alive at $K_{j}$ and are defined as

(9) $$\beta_{k}^{i,j}=\text{rank}(H_{k}^{i,j})=\text{rank}(Z_{k}(K_{i})∕(B_{k}(K_{j})\cap Z_{k}(K_{i}))).$$

**Figure 2:**
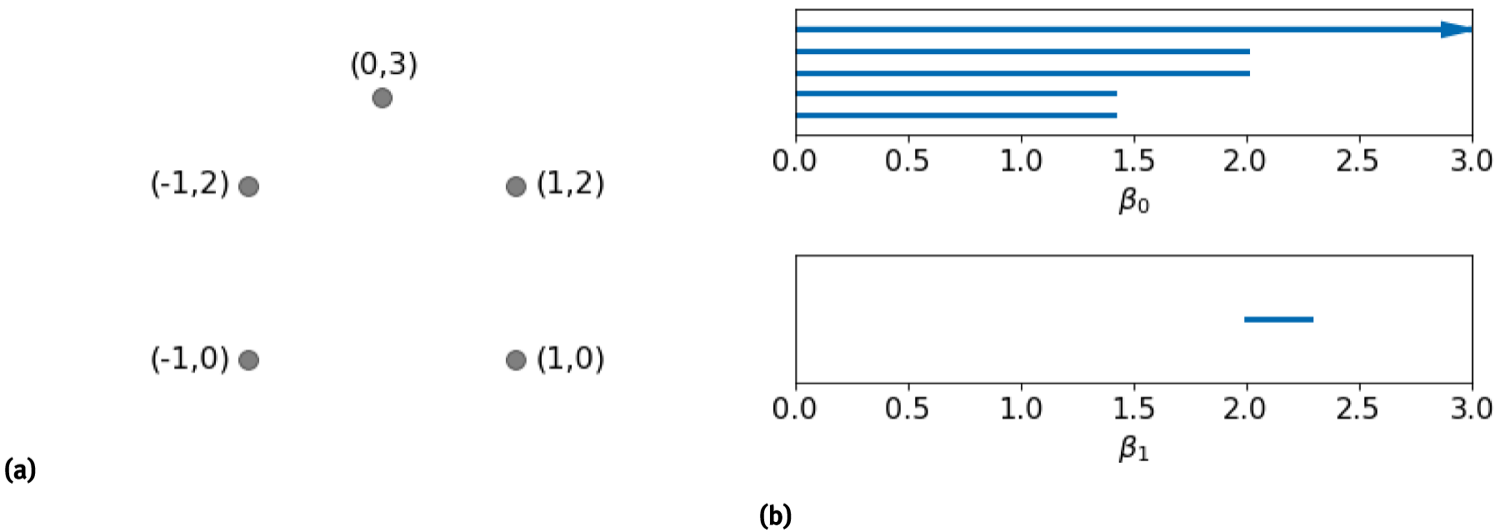
(a) An example of 5 points in $R^{2}$ and (b) the corresponding persistence barcodes. The length of each barcode corresponds to the persistence of each topological object (*β*_0_, *β*_1_, *β*_2_, etc..) over the Vietoris-Rips (VR) complex filtration.

The persistence of Betti numbers over the filtration interval can be recorded in many different ways. The commonly used ones are persistence barcodes and persistence diagrams. An example of barcodes is provided in Figure 2.

#### 2.1.5 Similarity and distance

In this work, we use Bottleneck and Wasserstein distances to extract atom-specific topological information and facilitate atom-specific persistent homology. Let *X* and *Y* be multisets of data points, the Bottleneck and Wasserstein distances of *X* and *Y* are given by [15]

(10) $$d_{B}(X,Y)=\underset{\gamma \in B(X,Y)}{\text{inf}}\underset{x\in X}{\text{sup}}\|x-\gamma (x){\|}_{\infty },$$

and [14]

(11) $$d_{W}^{p}(X,Y)={\left(\underset{\gamma \in B(X,Y)}{\text{inf}}\sum_{x\in X}\|x-\gamma (x){\|}_{\infty }^{p}\right)}^{1∕p},$$

respectively. Here *B*_(_*X*, *Y*) is the collection of all bijections from *X* to *Y*. Note that in our work, topological invariants of different dimensions are compared separately.

#### 2.1.6 Vietoris-Rips complex

Given a metric space *M* and a cutoff distance *d*, a simplex is formed if all points have pairwise distances no greater than *d*. All such simplices form the Vietoris-Rips (VR) complex. The abstract nature of the VR complex allows the construction of simplicial complexes from a correlation function, which models the pairwise interaction of atoms using a radial basis function versus more standard distance metrics. The R library TDA is used to generate persistence barcodes [37].

#### 2.1.7 Atom-specific persistent homology and element-specific persistent homology

Element-specific persistent homology was introduced to embed chemical and biology information into topological invariants [12, 19]. Its essential idea is to construct topological representations from subsets of atoms in various element types in a protein. For example, if one selects all carbon atoms in a protein, the resulting persistence barcodes will represent the strength and network of hydrophobicity in the protein.

**Figure 3:**
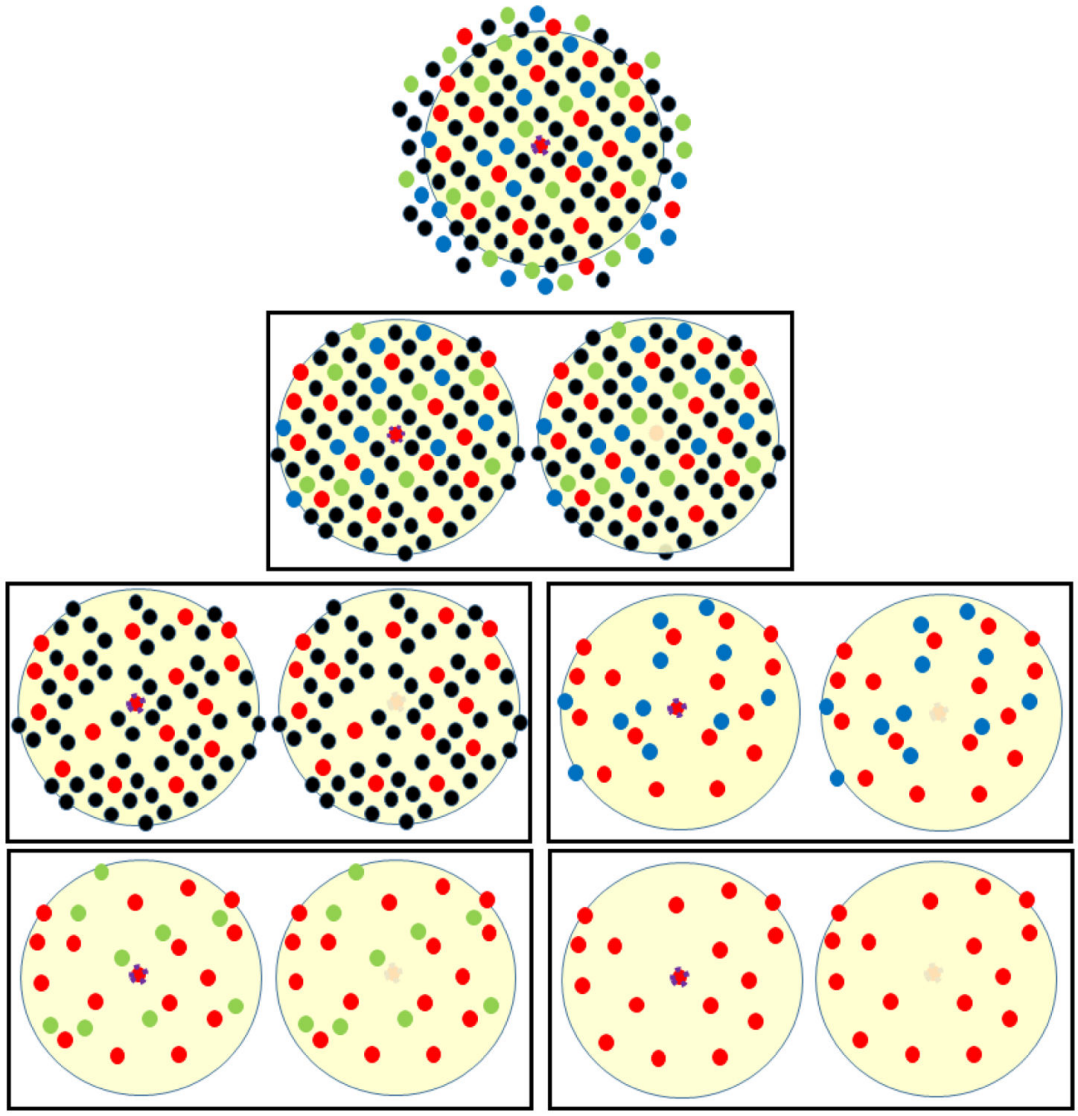
Illustration of Atom-specific persistent homology point clouds. Top: the original point cloud. The atom of interest is at the center of the circle. Second row: a pair of conjugated sets of point clouds for atom-specific persistent homology. The rest: Four pairs of conjugated point clouds for atom-specific and element-specific persistent homology.

In contrast, atom-specific persistent homology is designed to highlight the topological information of a given atom in a biomolecule. It creates two conjugated subsets of atoms centered around the atom of interest, one with and one without the specific atom. Conjugated simplicial complexes, conjugated homology groups and conjugated topological invariants are generated for the conjugated sets of points clouds. The difference between the conjugated topological invariants, measured by both Wasserstein and Bottleneck distances, offers a topological representation of the atom of interest. As shown in Figure 3, atom-specific and element-specific conjugated point clouds can be constructed for a given dataset.

In this work, we focus on C_*α*_ B-factor predictions. We use element specific persistent homology to enhance the topological representation of each C_*α*_ neighborhood. Meanwhile, we develop atom-specific persistent homology to pinpoint the topological representation at each C_*α*_ atom. With these selections of subsets, Vietoris-Rips complexes are constructed by contact maps or matrix filtration [1].

To capture element-specific interactions we consider three subsets of carbon-carbon, carbon-nitrogen, and carbon-oxygen point clouds. This gives us the following element specific pairs,

(12) P={CC,CN,CO}.

**Figure 4:**
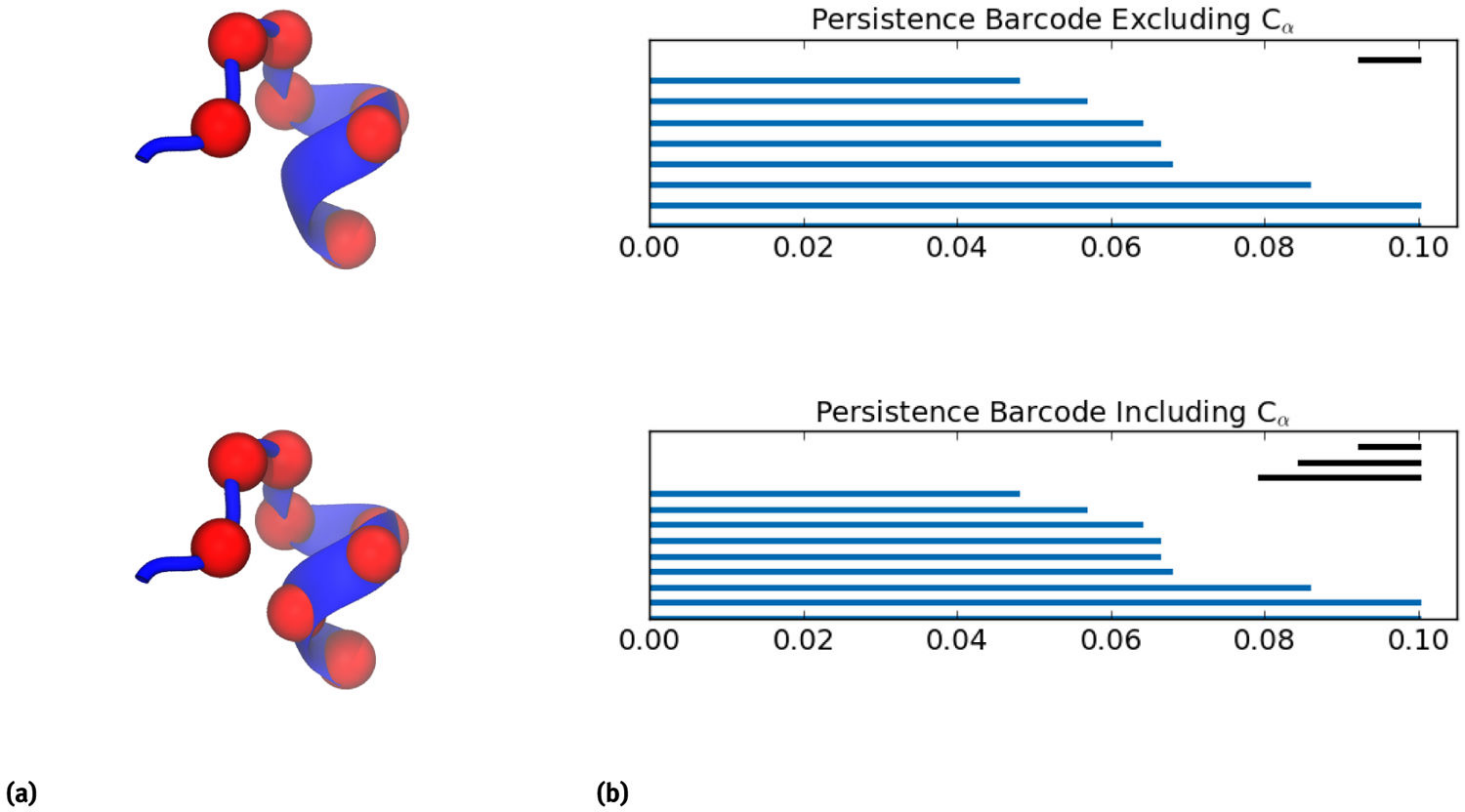
Illustration of atom-specific persistent homology using the fragments of protein 1AIE near residue 338 (i.e., residues 332-339). The left chart provides illustrations of the protein with and without C_*α*_ 338 from residue 338. The right chart shows conjugated persistence barcodes generated with and without C_*α*_ 338.

For a given Protein Data Bank (PDB) file, persistence barcodes are calculated as follows. Given a specific C_*α*_ of interest, say $r_{i}^{k}\in P_{k}$ in an element specific set $P_{k}$ ($P_{1}=\text{CC}$, $P_{2}=\text{CN}$, and $P_{3}=\text{CO}$), a point cloud consisting of all atoms within a pre-defined cutoff radius *r_c_* is selected:

(13) $$R_{i}^{k}=\{r_{j}^{k}\;|\;\|r_{i}^{k}-r_{j}^{k}\|<r_{c},\;\;r_{i}^{k},r_{j}^{k}\in P_{k},\forall j\in 1,2,\ldots N\},$$

where *N* is the number of atoms in the *k*th element pair $P_{k}$. A conjugated set of point cloud, ${\hat{R}}_{i}^{k}$, includes the same set of atoms, except for $r_{i}^{k}$. For a given pair of conjugated point clouds $R_{i}^{k}$ and ${\hat{R}}_{i}^{k}$, conjugated simplicial complexes, conjugated homology groups, and conjugated persistence barcodes are computed via persistent homology. We compute Euclidean distance based filtration using the Vietoris-Rips complex. Additionally, for a given set of atoms selected according to atom-specific and element specific constructions, we generate a family of multiresolution persistence barcodes by a resolution controlled filtration matrix: [1]

(14) $$M_{nm}(\vartheta )=1-\Phi (\|r_{n}-r_{m}\|;\vartheta ),$$

where *ϑ* denotes a set of kernel parameters. We have used both exponential kernels

(15) $$\Phi (\|r_{n}-r_{m}\|;\eta ,\kappa )=e^{-(\|r_{n}-r_{m}\|∕\eta {)}^{\kappa }},\;\;\;\kappa >0,$$

and Lorentz kernels

(16) $$\Phi (\|r_{n}-r_{m}\|;\eta ,v)=\frac{1}{1+(\|r_{n}-r_{m}\|∕\eta {)}^{v}},\;\;\;v>0,$$

where *η κ*, and *ν* are pre-defined constants. This filtration matrix is used in association with the Vietoris-Rips complex to generate persistence barcodes or persistence diagrams. Then these topological invariants are compared using both Bottleneck and Wasserstein distances. An example of the conjugated persistence barcode pair generated for a C_*α*_ atom is illustrated in Figure 4.

### 2.2 Machine learning models

Topological features are used for prediction of protein B-factor using both least squares fitting and machine learning as described in the following subsections.

### 2.2.1 Gradient boosted trees

Gradient boosting is an ensemble method that uses a number of “weak learners” to construct a prediction model in an iterative manner. The method is optimized via gradient descent, which minimizes the residuals of a loss function. At each step of the gradient boosting, gradient boosting trees (GBTs) incorporate decision trees to improve their predictive power. Ensemble methods like GBTs are useful because they can handle a diverse feature set, have strong predictive power, and are typically robust to outliers and against overfitting.

In this work, we optimize the GBT hyper-parameters using the standard practice of a grid search. The parameters used for testing are provided in Table 2. Any hyper-parameters not listed in the table were taken to be the default values provided by the python scikit-learn package (version 0.21.3).

#### 2.2.2 Deep learning with a convolutional neural network

Neural networks are modeled after the function of neurons in brain. A neural network applies activation functions, called perceptrons, to inputs. Weights of the network are trained to minimize a loss function over many epochs, or passes of an entire training dataset. When a neural network has several layers of perceptrons we call it a deep neural network (DNN) and the intermediate layers are known as hidden layers.

**Figure 5:**
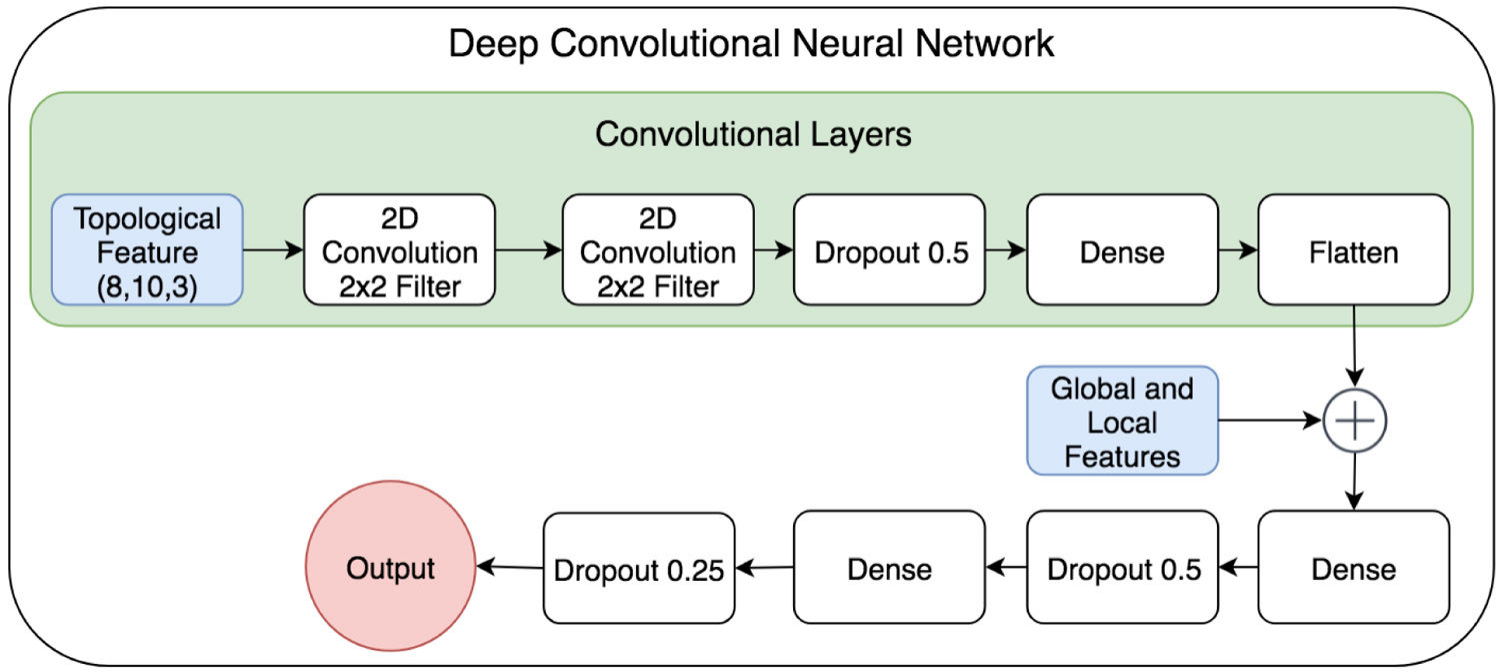
The deep learning architecture using a convolutional neural network combined with a deep neural network. The plus symbol represents the concatenation of features.

Convolutional neural networks (CNNs) have recently had great success in image classification. Using convolutions of a pre-defined filter size and number of filters, CNNs can automatically extract high-level features from input images. CNNs are advantageous because they can perform as well as other models without training as many parameters as a densely connected deep neural network. By applying several convolutions one can extract high-level features of an image. In this work we generate a image-like heat map by using a range of kernel parameters for atom-specific and element-specific persistent homology. The CNN output is then flattened and fed as input to a DNN along with global and local protein features. This allows us to use the same feature set as the boosted gradient method as well as the generated PH image data. A diagram of the CNN architecture is provided in Figure 5.

For each C_*α*_ of the training set, the CNN is passed a three-channel persistent homology image of dimension (8,10,3). The model takes the input image data and applies two convolutional layers with 2x2 filters followed by a dropout of 0.5. The image data is passed through a dense layer, flattened, then joined with the other global and local features to form a dense layer of 218 neurons. This is followed by a dropout layer of 0.5, another dense layer of 100 neurons, a dropout layer of 0.25, a dense layer of 10 neurons, and finishes with a dense layer of output. Figure 5 provides an illustration of the deep CNN used in this work.

The deep convolutional neural network has several hyper-parameters that can be tuned. As with the GBT, the deep convolutional neural network hyper-parameters are optimized using a basic grid search. Table 3 provides the parameters used for testing. Any hyper-parameters that are not listed below were taken to be the default values provided by the python Keras package.

#### 2.2.3 Consensus method

In this work, we combine the predictions of two machine learning models to construct a simple consensus model. The consensus prediction used in this work is generated by the average of C_*α*_ B-factor values predicted from the GBT and deep CNN models.

### 2.3 Machine learning features

A variety of element-specific and atom-specific persistence barcodes were generated using the techniques discussed in Sec. 2.1.7. In this work, we include 60 topological features. These features are generated in several ways by varying: kernels (Lorentz and exponential), element-specific pairs (CC, CN, CO), and distance metrics (Wasserstein-0 and Wasserstein-1, Bottleneck-0 and Bottleneck-1). For this work all persistent homology features were generated with the cutoff of 11Å.

#### 2.3.1 Wasserstein and Bottleneck metrics for modified persistence diagrams

**Figure 6:**
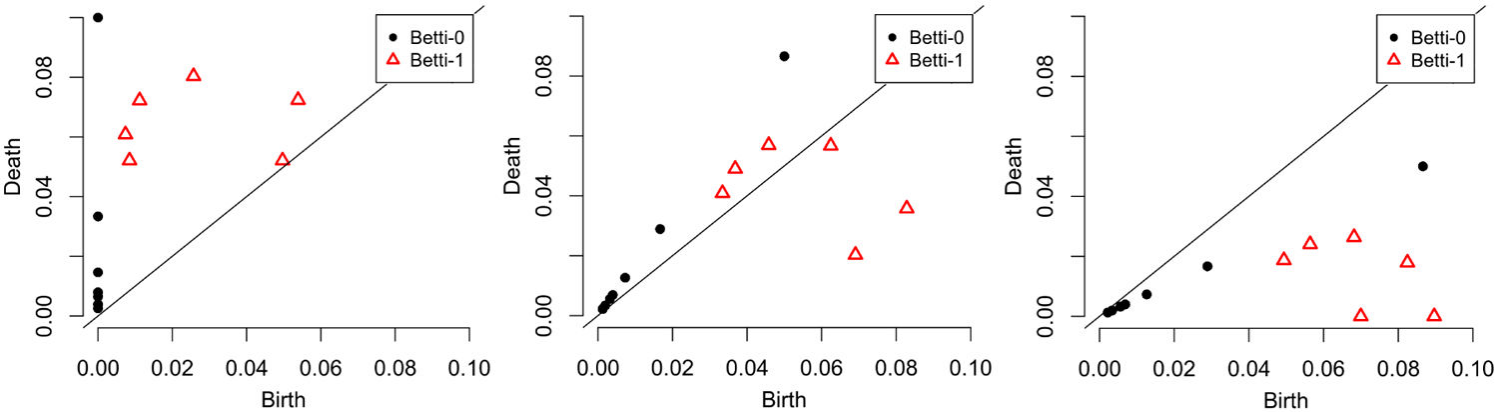
Illustration of modified persistence diagrams used in distance calculations. Left: Unchanged. Middle: Rotated 30°. Right: rotated 60°. Black dots are Betti-0 events and triangles are Betti-1 events.

The distances evaluated from Wasserstein and Bottleneck evaluations of persistence diagrams depend on the boundary of the diagrams. Specifically, when two persistence diagrams are compared, the extra events on one diagram that do not match any events on the other diagram might contribute to the final distance by their distances from the boundary. For this reason, we create two additional persistence diagrams in which the *y*-axis is rotated clockwise by 30° or 60°, respectively, see Figure 6. This modification changes the Bottleneck and Wasserstein distances and allows the model to recognize elements that have a short persistence (i.e. have a short lifespan). Lastly, we modified the persistence diagram by reflecting around the diagonal axis. An example of this modification is illustrated in Figure 6. Table 4 provides a list of kernels, kernel parameters, *y*-axis change, distance metric, and element-specific pairs used to generate features in machine learning models.

Other features include global features from PDB files, i.e., R-value, protein resolution, and number of heavy atoms. Additional local features include packing density, amino acid type, occupancy, and secondary structure information generated by STRIDE software [38].

#### 2.3.2 Image-like persistent homology features

Using the process described in Section 2.1.7 we generate 2D image-like persistent homology features, $F_{i}^{k}=\{f_{i}^{k}(\eta ,\kappa \}$, for each C_*α*_ of the proteins in the dataset by using various values of *η* and *κ* in the kernel function. A cutoff of 11 Å with an exponential kernel and different values of *η* and *κ* are used to capture a wide variety of scales. In particular we use

*η* = {1, 2, 3, 4, 5, 10, 15, 20},

and

*κ* = {1, 2, 3, 4, 5, 6, 7, 8, 9, 10}.

The image-like matrix is given by $F_{i}^{k}$ in Eq. (17), where each atom $F_{i}^{k}$ represents the PH feature of the *i^th^* C_*α*_ atom, and *k^th^* atom interaction (C, N, or O).

(17) Fik={[fik(1,1)fik(1,2)⋯fik(1,9)fik(1,10)fik(2,1)fik(2,2)⋯fik(2,9)fik(2,10)⋮⋮fik(15,1)fik(15,2)⋯fik(15,9)fik(15,10)fik(20,1)fik(20,2)⋯fik(20,9)fik(20,10)︸κ]}η

This results in 2D PH images of dimension (8,10). Images are created for element-specific C_*α*_ interactions with carbon, nitrogen, and oxygen atom giving each image three channels. This results in a final image dimension of (8,10,3) for each C_*α*_ atom.

## 3 Results

### 3.1 Data sets

In this work, we use two data sets, one from Refs. [32, 33] and the other from Park, Jernigan, and Wu [39]. The first contains 364 proteins [32, 33] and the second contains 3 subsets of small, medium, and large proteins [39]. All sequences have a resolution of 3 Å or higher and an average resolution of 1.3 Å and the sets include proteins that range from 4 to 3912 residues [39].

For all testing, we exclude protein 1AGN due to known problems with this protein data [33]. Proteins 1NKO, 2OCT, and 3FVA are also excluded because these proteins have residues with B-factors reported as zero, which is unphysical. For the machine learning results, proteins 1OB4, 1OB7, 2OLX, and 3MD5 are excluded because the STRIDE software is unable to provide secondary features for these proteins. The image like features used in all convolutional neural networks were standardized with mean 0 and variance of 1

### 3.2 Evaluation metric

We use the proposed methods to predict B-factors of all C_*α*_ atoms present in a protein. Linear least square fitting was done using only topological features. The machine learning models were executed using a leave-one-(protein)-out method to blindly predict the B-factors of all C_*α*_ atoms in each protein. The machine learning models were trained using the data and features described in Sections 2.1.7, 2.2, 2.3. For comparison, we include previously existing C_*α*_ B-factor prediction fitting methods.

To quantitatively assess our method for B-factor prediction we use the Pearson correlation coefficient given by

(18) $$\text{PCC}=\frac{\sum_{i=1}^{N}(B_{i}^{e}-{\bar{B}}^{e})(B_{i}^{t}-{\bar{B}}^{t})}{{\left[\sum_{i=1}^{N}(B_{i}^{e}-{\bar{B}}^{e}{)}^{2}\sum_{i=1}^{N}(B_{i}^{t}-{\bar{B}}^{t}{)}^{2}\right]}^{1∕2}},$$

where $B_{i}^{t}$, *i* = 1, 2,…, *N* are predicted B-factors using the proposed method and $B_{i}^{e}$, *i* = 1, 2,…, *N* experimental B-factors from the PDB file. The terms $B_{i}^{t}$ and $B_{i}^{e}$ represent the *i^th^* theoretical and experimental B-factors respectively. Here ${\bar{B}}^{e}$ and ${\bar{B}}^{t}$ are averaged B-factors.

### 3.3 Cutoff distance

**Figure 7:**
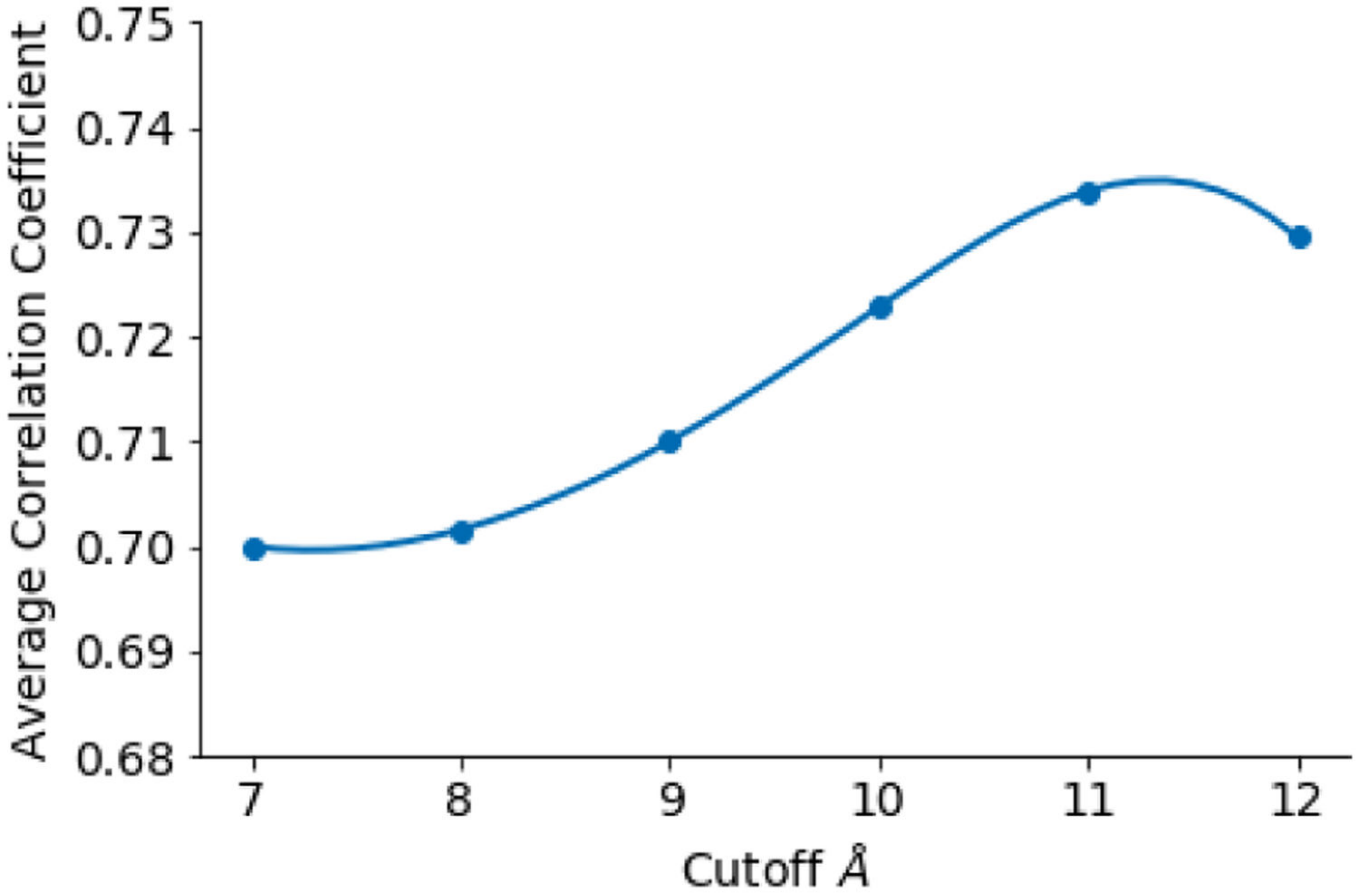
Average pearson correlation coefficient over the entire protein dataset fitting all 24 persistent homology features using various cuttoff distances.

In this work, the optimal cutoff of *r_c_* = 11Å is found over a grid search using various cutoff distances. Figure 7 displays the average Pearson correlation coefficient, obtained via fitting, over an entire dataset of 364 protein using all persistent homology metrics with various point cloud distance cutoffs.

For each protein we use the parameters listed in Table 5. The values used in this work were determined using the standard practice of a grid search.

### 3.4 Least squares fitting within proteins

The Pearson correlation coefficients using least squares fitting for C_*α*_ B-factor prediction of small, medium, and large protein subsets are provided in Tables 12, 13, and 14 respectively. Results for the all proteins in the dataset are provided in Table 15. The average Pearson correlation coefficients for small, medium, large, and superset data sets are provided in Table 6. Table 6 includes fitting results using only Bottleneck, only Wasserstein, and using both Bottleneck and Wasserstein metrics. We also include results using only exponential kernel, only a Lorentz kernel, or both an exponential and Lorentz kernel for fitting. All results reported here PH features generated with a cutoff of 11Å and include three element-specific subsets (carbon-carbon, carbon-nitrogen, carbon-oxygen). Overall fitting methods using the various persistent homology features performed similarly. The best results came from using features generated by both exponential and Lorentz kernels and both Bottleneck and Wasserstein distances. Using both kernels and both distance metrics resulted in an average correlation coefficient of 0.73 for the superset.

### 3.5 Blind machine learning prediction across proteins

The aforementioned least squares fitting methods cannot predict the B-factors of unknown proteins. Machine learning methods enable us to blindly predict B-factors across proteins. In this section, we utilize both boosted gradient and convolutional neural network algorithms for the blind prediction of B-factor across different proteins. Taken together, the entire dataset contains more than 620 000 atoms. We use a leave-one-protein out cross validation in our prediction. That is, for each protein, the data from a protein whose B-factors will be predicted, is excluded from the training data. This gives rise to a training set of roughly 600 000 data points for each protein (i.e., atoms and associated B-factors). The Pearson correlation coefficients using boosted gradient (GBT), convolutional neural network (CNN), and consensus method (CON) for C_*α*_ B-factor prediction of small, medium, and large protein subsets are provided in Tables 8, 9, and 10 respectively. Parameters for GBT and CNN methods can be found in Tables 2 and 3. The global and local features used for training and testing are provided in Section 2.3. Results for all proteins are provided in Table 11. The average Pearson correlation coefficients for small, medium, large, and superset data sets are provided in Table 7. All results reported here use a cutoff of 11Å and include three element-specific subsets (carbon-carbon, carbon-nitrogen, carbon-oxygen). Kernel parameters for both exponential and Lorentz kernels are provided in Table 5. Results from previously existing C_*α*_ B-factor prediction methods are included for comparison in Table 7. Overall both GBT and CNN algorithms perform similarly. As expected, the CNN method outperforms the GBT with average correlation coefficients over the superset of 0.60 and 0.59, respectively. The consensus method improves upon both results with an average Pearson correlation coefficient of 0.61 over the superset. Table 7 shows that the blind prediction machine learning models perform better than fitting models GNM and NMA and similar to the pFRI fitting model.

## 4 Conclusion

An essential component of the paradigm of protein dynamics is the correlation between protein flexibility and protein function. The shear complexity and large number of degrees of freedom make quantitative understanding of flexibility and function an inherently difficult problem. Several time-independent methods for predicting protein B-factors exist. Examples include NMA [24, 40, 25, 23], ENM [26], GNM [28, 29, 41], and FRI methods [31, 32, 33, 42]. None of the methods above are able to blindly predict protein B-factors of an unknown protein. We hypothesize that the intrinsic physics of proteins lie in a low-dimensional space embedded in a high-dimensional data space. Based on this hypothesis the authors previously introduced the graph theory based multiscale weighted colored graph (MWCG) [34, 35]. The authors showed that MWCG’s are able to successfully blindly predict cross-protein B-factors.

In this work we explore this hypothesis further by creating a B-factor predictor using tools from algebraic topology. In order to construct localized topological representations for individual atoms from global topological tools, we propose atom-specific topology and atom-specific persistent homology. This approach creates two conjugated sets of atoms: the first set is centered around the given atom of interest while the other set is identical but excludes the atom of interest. Element-specific selections are further implemented to embed biological information into atom-specific persistent homology. The distance between the topological invariants generated from these conjugated sets of atoms is used to represent the atom of interest. Both Bottleneck and Wasserstein metrics are utilized to estimate the topological distances between conjugated barcodes. The Vietoris-Rips complex is employed for topological barcode generation.

To test the proposed method we use over 300 proteins or more than 600,000 B-factors. Atom-specific persistent homology features are generated using several element-specific interactions, kernel choices, parametrizations, and barcode distance metrics. First we employ topological features to fit protein B-factors using linear least squares. Using topological features our fitting model outperformed previous fitting models with an average Pearson correlation coefficient of 0.73 over the superset of proteins. Next we considered using the topological features to blindly predict protein B-factors of C_*α*_ atoms. We generated two machine learning models, a gradient boosted tree (GBT) and deep convolutional neural network (CNN). Additionally we averaged the C_*α*_ prediction from the two models to generate a more robust consensus model. A variety of local and global features were included in addition to the generated topological features. Our blind prediction consensus model outperformed both GNM and NMA fitting models and produced results similar to those of the pFRI fitting model.

To the authors’ knowledge, this work is the first time persistent homology has been used to predict the B-factor of atoms in proteins. This approach is novel because topology is a global property and on its own cannot be directly used to describe local atomic information. Our unique approach allows us to create a localized topological representation using a global mathematical tool. This approach enables us to account for multiple spatial interaction scales and element specific interactions. Our results demonstrate that this is an accurate and robust topological approach. Moreover, the results could easily be improved by including a larger dataset, fine tuning parameters, and exploring different machine learning algorithms.

This method can be applied to a variety of interesting applications related to molecules and biomolecules. Examples include allosteric site detection, hinge detection, hot spot identification, chemical shift analysis, atomic spectroscopy interpretation, and prediction of protein folding stability changes upon mutation. More generally this method may be amenable to problems outside chemistry and biology such as network dynamics and social network centrality measure.

## Figures and Tables

**Table 1:** Topological invariants displayed as Betti numbers. Betti-0 represents the number of connected components, Betti-1 the number of tunnels or circles, and Betti-2 the number of cavities or voids. Two auxiliary rings are added to the torus to illustrate that its Betti-1=2.

	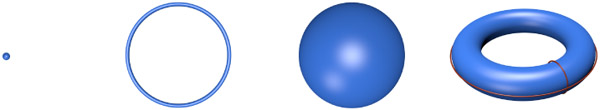			
Example	Point	Circle	Sphere	Torus
Betti-0	1	1	1	1
Betti-1	0	1	0	2
Betti-2	0	0	1	1

**Table 2:** Boosted gradient tree hyperparameters used for testing. Parameters were determined using a grid search. Any hyperparameters that is not listed were taken to be the default values provided by the python scikit-learn package.

Parameter	Setting
Loss Function	Quantile
Alpha	0.975
Estimators	500
Learning Rate	0.25
Max Depth	4
Min Samples Leaf	9
Min Samples Split	9

**Table 3:** Convolutional Neural Network (CNN) parameters used for testing. Parameters were determined using a grid search. Any hyper-parameters not listed below were taken to be the default values provided by python with the Keras package.

Parameter	Setting
Learning Rate	0.001
Epoch	1000
Batch Size	1000
Loss	Mean Squared Error
Optimizer	Adam

**Table 4:** Parameters used for topological feature generation. All features used a cutoff of 11Å. Both lorentz (Lor) and exponential (exp) kernels and Bottleneck (B) and Wasserstein (W) distance metrics were used.

No. features	Kernel	Kernel parameter	Diagram	Distance metric	Element-specific pair
12	Lor	*η* = 21, *ν* = 5	Unchanged	B, W	CC, CN, CO
12	Exp	*η* = 10, *κ* = 1	Unchanged	B, W	CC, CN, CO
12	Exp	*η* = 2, *κ* = 1	Diagonal reflection	B, W	CC, CN, CO
12	Exp	*η* = 2, *κ* = 1	Rotated 30°	B, W	CC, CN, CO
12	Exp	*η* = 2, *κ* = 1	Rotated 60°	B, W	CC, CN, CO

**Table 5:** Parameters used for the persistent homology element specific features with a cutoff of 11 Å.

Kernel Type	*ν*	*η*	*κ*
Lorentz	5	21	-
Exponential	-	10	1

**Table 6:** Average Pearson correlation coefficients of least squares fitting C_*α*_ B-factor prediction of small, medium, large, and superset using 11Å cutoff. Two Bottleneck (B) and Wasserstein (W) metrics using various kernel choices are included. Results for pFRI are taken from Opron et al[32]. GNM and NMA value are taken from the course grained C_*α*_ results reported in Park *et al* [39].

H												
	B & W			B			W			pFRI	GNM	NMA
	Exp	Lor	Both	Exp	Lor	Both	Exp	Lor	Both			
Small	0.87	0.84	0.94	0.74	0.72	0.85	0.74	0.73	0.86	0.59	0.54	0.48
Medium	0.68	0.68	0.78	0.62	0.61	0.69	0.60	0.63	0.69	0.61	0.55	0.48
Large	0.61	0.60	0.70	0.54	0.54	0.61	0.51	0.55	0.62	0.59	0.53	0.49
Superset	0.65	0.64	0.73	0.58	0.58	0.65	0.55	0.59	0.66	0.63	0.57	NA

**Table 7:** Average Pearson correlation coefficients C_*α*_ B-factor predictions for small-, medium-, and large-sized protein sets along with the entire superset of the 364 protein dataset. Gradient boosted tree (GBT), convolutional neural network, and consensus (CON) results are obtained by leave-one-protein-out (blind). The results of parameter-free flexibility-rigidity index (pf-FRI), Gaussian network model (GNM) and normal mode analysis (NMA) were obtained via the least squares fitting of individual proteins.

	CNN	GBT	CON	pFRI	GNM	NMA
Small	0.63	0.58	0.62	0.59	0.54	0.48
Medium	0.60	0.58	0.61	0.61	0.55	0.48
Large	0.58	0.59	0.58	0.59	0.53	0.49
Superset	0.60	0.59	0.61	0.63	0.57	NA

## Appendix

**Table 8:** Pearson correlation coefficients for cross protein C_*α*_ atom blind B-factor prediction obtained by boosted gradient (GBT), convolutional neural network (CNN), and consensus (CON) for the small-sized protein set.

PDB ID	*N*	GBT	CNN	CON
1AIE	31	0.75	0.7	0.78
1AKG	16	0.27	0.32	0.29
1BX7	51	0.74	0.74	0.76
1ETL	12	0.37	0.82	0.55
1ETM	12	0.37	0.63	0.43
1ETN	12	0.07	0.48	0.13
1FF4	65	0.61	0.66	0.64
1GK7	39	0.77	0.9	0.82
1GVD	56	0.71	0.55	0.69
1HJE	13	0.84	0.75	0.9
1KYC	15	0.62	0.69	0.66
1NOT	13	0.69	0.96	0.8
1O06	22	0.94	0.93	0.95
1P9I	29	0.73	0.73	0.74
1PEF	18	0.79	0.82	0.82
1PEN	16	0.36	0.74	0.44
1Q9B	44	0.59	0.85	0.67
1RJU	36	0.6	0.46	0.58
1U06	55	0.44	0.4	0.45
1UOY	64	0.72	0.7	0.76
1USE	47	0.05	0.32	0.12
1VRZ	13	0.54	0.34	0.54
1XY2	8	0.79	0.82	0.81
1YJO	6	0.7	−0.06	0.57
1YZM	46	0.69	0.64	0.7
2DSX	52	0.34	0.34	0.36
2JKU	38	0.57	0.71	0.66
2NLS	36	0.23	0.47	0.29
2OL9	6	0.94	0.85	0.94
6RXN	45	0.59	0.6	0.61

**Table 9:** Pearson correlation coefficients for cross protein C_*α*_ atom blind B-factor prediction obtained by boosted gradient (GBT), convolutional neural network (CNN), and consensus (CON) for the medium-sized protein set.

PDB ID	*N*	GBT	CNN	CON
1ABA	87	0.73	0.71	0.74
1CYO	88	0.64	0.7	0.68
1FK5	93	0.59	0.6	0.61
1GXU	89	0.67	0.68	0.69
1I71	83	0.53	0.58	0.56
1LR7	73	0.62	0.61	0.64
1N7E	95	0.63	0.58	0.65
1NNX	93	0.78	0.79	0.8
1NOA	113	0.55	0.53	0.56
1OPD	85	0.42	0.34	0.41
1QAU	112	0.51	0.59	0.57
1R7J	90	0.71	0.77	0.75
1UHA	82	0.71	0.74	0.73
1ULR	87	0.54	0.53	0.56
1USM	77	0.73	0.72	0.75
1V05	96	0.6	0.64	0.63
1W2L	97	0.43	0.5	0.47
1X3O	80	0.41	0.43	0.44
1Z21	96	0.68	0.65	0.69
1ZVA	75	0.7	0.7	0.71
2BF9	35	0.48	0.79	0.58
2BRF	103	0.72	0.77	0.75
2CE0	109	0.6	0.66	0.64
2E3H	81	0.65	0.68	0.67
2EAQ	89	0.57	0.63	0.61
2EHS	75	0.62	0.67	0.65
2FQ3	85	0.77	0.82	0.81
2IP6	87	0.6	0.66	0.63
2MCM	112	0.71	0.77	0.75
2NUH	104	0.72	0.56	0.7
2PKT	93	0.01	−0.04	−0.01
2PLT	98	0.52	0.53	0.54
2QJL	107	0.54	0.57	0.56
2RB8	93	0.67	0.7	0.7
3BZQ	99	0.45	0.53	0.49
5CYT	103	0.39	0.34	0.39

**Table 10:** Pearson correlation coefficients for cross protein C_*α*_ atom blind B-factor prediction obtained boosted gradient (GBT), convolutional neural network (CNN), and consensus (CON) for the large-sized protein set.

PDB ID	*N*	GBT	CNN	CON
1AHO	66	0.66	0.66	0.7
1ATG	231	0.55	0.51	0.55
1BYI	238	0.61	0.5	0.6
1CCR	109	0.55	0.6	0.59
1E5K	188	0.74	0.72	0.74
1EW4	106	0.59	0.6	0.61
1IFR	113	0.7	0.64	0.7
1NLS	238	0.55	0.57	0.57
1O08	221	0.49	0.47	0.49
1PMY	123	0.59	0.7	0.65
1PZ4	113	0.72	0.8	0.77
1QTO	122	0.53	0.48	0.54
1RRO	108	0.4	0.45	0.43
1UKU	102	0.75	0.76	0.77
1V70	105	0.63	0.62	0.64
1WBE	206	0.6	0.56	0.6
1WHI	122	0.59	0.56	0.6
1WPA	107	0.65	0.65	0.67
2AGK	233	0.67	0.63	0.67
2C71	225	0.57	0.6	0.6
2CG7	110	0.3	0.32	0.32
2CWS	235	0.61	0.47	0.6
2HQK	232	0.77	0.77	0.78
2HYK	237	0.65	0.63	0.65
2I24	113	0.44	0.46	0.46
2IMF	203	0.53	0.58	0.56
2PPN	122	0.64	0.54	0.63
2R16	185	0.44	0.49	0.46
2V9V	149	0.53	0.52	0.54
2VIM	114	0.44	0.47	0.47
2VPA	217	0.66	0.75	0.71
2VYO	207	0.6	0.63	0.63
3SEB	238	0.63	0.6	0.63
3VUB	101	0.59	0.55	0.59

**Table 11:** Pearson correlation coefficients for cross protein C_*α*_ atom blind B-factor prediction obtained by boosted gradient (GBT), convolutional neural network (CNN), and consensus method (CON) for the Superset.

PDB ID	*N*	GBT	CNN	CON	PDB ID	*N*	GBT	CNN	CON
1ABA	87	0.73	0.71	0.74	2X5Y	185	0.76	0.68	0.76
1AHO	66	0.66	0.66	0.7	2X9Z	266	0.49	0.52	0.52
1AIE	31	0.75	0.7	0.78	2XHF	310	0.58	0.57	0.58
1AKG	16	0.27	0.32	0.29	2Y0T	111	0.71	0.71	0.74
1ATG	231	0.55	0.51	0.55	2Y72	183	0.65	0.71	0.69
1BGF	124	0.61	0.58	0.62	2Y7L	*323*	0.66	0.66	0.68
1BX7	51	0.74	0.74	0.76	2Y9F	149	0.74	0.75	0.76
1BYI	238	0.61	0.5	0.6	2YLB	418	0.67	0.66	0.7
1CCR	109	0.55	0.6	0.59	2YNY	326	0.65	0.71	0.69
1CYO	88	0.64	0.7	0.68	2ZCM	348	0.33	0.38	0.36
1DF4	57	0.85	0.85	0.88	2ZU1	360	0.66	0.66	0.68
1E5K	188	0.74	0.72	0.74	3A0M	146	0.53	0.6	0.59
1ES5	260	0.65	0.62	0.66	3A7L	128	0.44	0.61	0.53
1ETL	12	0.37	0.82	0.55	3AMC	614	0.68	0.64	0.69
1ETM	12	0.37	0.63	0.43	3AUB	124	0.5	0.5	0.55
1ETN	12	0.07	0.48	0.13	3B5O	249	0.49	0.55	0.52
1EW4	106	0.59	0.6	0.61	3BA1	312	0.62	0.59	0.63
1F8R	1932	0.52	0.54	0.54	3BED	262	0.45	0.53	0.5
1FF4	65	0.61	0.66	0.64	3BQX	136	0.56	0.55	0.58
1FK5	93	0.59	0.6	0.61	3BZQ	99	0.45	0.53	0.49
1GCO	1044	0.47	0.47	0.5	3BZZ	103	0.38	0.51	0.44
1GK7	39	0.77	0.9	0.82	3DRF	567	0.51	0.45	0.52
1GVD	56	0.71	0.55	0.69	3DWV	359	0.63	0.55	0.63
1GXU	89	0.67	0.68	0.69	3E5T	268	0.44	0.48	0.46
1H6V	2927	0.26	0.34	0.34	3E7R	40	0.72	0.66	0.77
1HJE	13	0.84	0.75	0.9	3EUR	150	0.36	0.42	0.38
1I71	83	0.53	0.58	0.56	3F2Z	148	0.73	0.76	0.75
1IDP	441	0.62	0.6	0.63	3F7E	261	0.65	0.69	0.68
1IFR	113	0.7	0.64	0.7	3FCN	185	0.63	0.65	0.66
1K8U	87	0.57	0.6	0.59	3FE7	89	0.52	0.55	0.54
1KMM	1499	0.64	0.51	0.63	3FKE	250	0.51	0.51	0.54
1KNG	144	0.5	0.52	0.51	3FMY	75	0.65	0.67	0.68
1KR4	107	0.56	0.71	0.63	3FOD	48	0.45	0.57	0.54
1KYC	15	0.62	0.69	0.66	3FSO	238	0.72	0.75	0.74
1LR7	73	0.62	0.61	0.64	3FTD	257	0.64	0.68	0.67
1MF7	194	0.65	0.66	0.67	3G1S	418	0.6	0.57	0.61
1N7E	95	0.63	0.58	0.65	3GBW	170	0.74	0.74	0.75
1NKD	59	0.7	0.7	0.72	3GHJ	129	0.58	0.56	0.59
1NLS	238	0.55	0.57	0.57	3HFO	216	0.51	0.57	0.54
1NNX	93	0.78	0.79	0.8	3HHP	1314	0.61	0.65	0.65
1NOA	113	0.55	0.53	0.56	3HNY	170	0.61	0.6	0.62
1NOT	13	0.69	0.96	0.8	3HP4	201	0.56	0.58	0.58
1O06	22	0.94	0.93	0.95	3HWU	155	0.58	0.65	0.62
1O08	221	0.49	0.47	0.49	3HYD	8	0.99	0.74	0.99
1OPD	85	0.42	0.34	0.41	3HZ8	200	0.45	0.54	0.48
1P9I	29	0.73	0.73	0.74	3I2V	127	0.44	0.52	0.48
1PEF	18	0.79	0.82	0.82	3I2Z	140	0.6	0.6	0.6
1PEN	16	0.36	0.74	0.44	3I4O	154	0.62	0.72	0.66
1PMY	123	0.59	0.7	0.65	3I7M	145	0.44	0.57	0.49
1PZ4	113	0.72	0.8	0.77	3IHS	173	0.61	0.62	0.64
1Q9B	44	0.59	0.85	0.67	3IVV	168	0.83	0.82	0.84
1QAU	112	0.51	0.59	0.57	3K6Y	227	0.56	0.57	0.58
1QKI	3912	0.34	0.45	0.38	3KBE	166	0.56	0.64	0.6
1QTO	122	0.53	0.48	0.54	3KGK	190	0.76	0.8	0.78
1R29	122	0.56	0.59	0.59	3KZD	94	0.55	0.67	0.6
1R7J	90	0.71	0.77	0.75	3L41	219	0.61	0.64	0.64
1RJU	36	0.6	0.46	0.58	3LAA	176	0.35	0.49	0.42
1RRO	108	0.4	0.45	0.43	3LAX	118	0.74	0.69	0.74
1SAU	123	0.54	0.66	0.59	3LG3	846	0.45	0.51	0.5
1TGR	111	0.66	0.69	0.69	3LJI	270	0.57	0.55	0.58
1TZV	157	0.74	0.77	0.76	3M3P	244	0.53	0.59	0.57
1U06	55	0.44	0.4	0.45	3M8J	178	0.72	0.71	0.74
1U7I	259	0.71	0.74	0.74	3M9J	250	0.56	0.52	0.56
1U9C	220	0.57	0.59	0.59	3M9Q	190	0.4	0.48	0.45
1UHA	82	0.71	0.74	0.73	3MAB	180	0.63	0.63	0.65
1UKU	102	0.75	0.76	0.77	3MD4	13	0.88	0.96	0.96
1ULR	87	0.54	0.53	0.56	3MEA	170	0.62	0.63	0.63
1UOY	64	0.72	0.7	0.76	3MGN	277	0.08	0.09	0.09
1USE	47	0.05	0.32	0.12	3MRE	446	0.54	0.54	0.57
1USM	77	0.73	0.72	0.75	3N11	325	0.51	0.47	0.52
1UTG	70	0.62	0.64	0.66	3NE0	208	0.67	0.73	0.71
1V05	96	0.6	0.64	0.63	3NGG	97	0.72	0.75	0.75
1V70	105	0.63	0.62	0.64	3NPV	500	0.51	0.5	0.54
1VRZ	13	0.54	0.34	0.54	3NVG	6	0.51	0.63	0.71
1W2L	97	0.43	0.5	0.47	3NZL	70	0.56	0.58	0.57
1WBE	206	0.6	0.56	0.6	3O0P	197	0.68	0.72	0.71
1WHI	122	0.59	0.56	0.6	3O5P	147	0.6	0.59	0.61
1WLY	322	0.64	0.62	0.66	3OBQ	150	0.59	0.57	0.59
1WPA	107	0.65	0.65	0.67	3OQY	236	0.66	0.59	0.66
1X3O	80	0.41	0.43	0.44	3P6J	145	0.66	0.72	0.69
1XY1	16	0.82	0.75	0.83	3PD7	216	0.68	0.7	0.71
1XY2	8	0.79	0.82	0.81	3PES	166	0.56	0.54	0.57
1Y6X	86	0.5	0.46	0.51	3PID	387	0.48	0.3	0.45
1YJO	6	0.7	−0.06	0.57	3PIW	161	0.72	0.77	0.75
1YZM	46	0.69	0.64	0.7	3PKV	229	0.52	0.51	0.53
1Z21	96	0.68	0.65	0.69	3PSM	94	0.8	0.77	0.82
1ZCE	139	0.7	0.74	0.73	3PTL	289	0.53	0.55	0.55
1ZVA	75	0.7	0.7	0.71	3PVE	363	0.61	0.61	0.63
2A50	469	0.6	0.54	0.6	3PZ9	357	0.61	0.58	0.63
2AGK	233	0.67	0.63	0.67	3PZZ	12	0.94	0.85	0.93
2AH1	939	0.48	0.55	0.54	3Q2X	6	0.95	0.72	0.93
2B0A	191	0.62	0.59	0.63	3Q6L	131	0.47	0.53	0.52
2BCM	415	0.5	0.51	0.52	3QDS	284	0.62	0.62	0.63
2BF9	35	0.48	0.79	0.58	3QPA	212	0.55	0.67	0.59
2BRF	103	0.72	0.77	0.75	3R6D	222	0.65	0.74	0.69
2C71	225	0.57	0.6	0.6	3R87	148	0.47	0.45	0.48
2CE0	109	0.6	0.66	0.64	3RQ9	165	0.46	0.4	0.46
2CG7	110	0.3	0.32	0.32	3RY0	128	0.41	0.49	0.46
2COV	534	0.74	0.72	0.75	3RZY	151	0.65	0.62	0.66
2CWS	235	0.61	0.47	0.6	3S0A	132	0.53	0.49	0.54
2D5W	1214	0.54	0.64	0.59	3SD2	100	0.56	0.56	0.57
2DKO	253	0.78	0.78	0.8	3SEB	238	0.63	0.6	0.63
2DPL	565	0.41	0.36	0.42	3SED	126	0.53	0.52	0.55
2DSX	52	0.34	0.34	0.36	3SO6	157	0.65	0.65	0.66
2OCT	439	0.64	0.67	0.67	3SR3	657	0.5	0.46	0.5
2E3H	81	0.65	0.68	0.67	3SUK	254	0.58	0.59	0.6
2EAQ	89	0.57	0.63	0.61	3SZH	753	0.69	0.67	0.71
2EHP	246	0.66	0.62	0.67	3T0H	209	0.71	0.7	0.73
2EHS	75	0.62	0.67	0.65	3T3K	122	0.76	0.76	0.78
2ERW	53	0.12	0.24	0.16	3T47	145	0.51	0.62	0.57
2ETX	390	0.49	0.48	0.51	3TDN	359	0.47	0.49	0.49
2FB6	129	0.73	0.75	0.75	3TOW	155	0.61	0.63	0.63
2FG1	176	0.57	0.61	0.59	3TUA	226	0.62	0.56	0.63
2FN9	560	0.57	0.54	0.58	3TYS	78	0.66	0.74	0.72
2FQ3	85	0.77	0.82	0.81	3U6G	276	0.53	0.46	0.52
2G69	99	0.62	0.5	0.6	3U97	85	0.67	0.72	0.71
2G7O	68	0.72	0.86	0.8	3UCI	72	0.42	0.42	0.43
2G7S	206	0.55	0.58	0.58	3UR8	637	0.64	0.6	0.64
2GKG	150	0.56	0.64	0.59	3US6	159	0.61	0.63	0.64
2GOM	121	0.69	0.59	0.69	3V1A	59	0.57	0.27	0.55
2GXG	140	0.65	0.67	0.68	3V75	294	0.49	0.56	0.53
2GZQ	203	0.34	0.4	0.37	3VN0	193	0.85	0.85	0.86
2HQK	232	0.77	0.77	0.78	3VOR	219	0.47	0.48	0.48
2HYK	237	0.65	0.63	0.65	3VUB	101	0.59	0.55	0.59
2I24	113	0.44	0.46	0.46	3VVV	112	0.56	0.57	0.57
2I49	399	0.65	0.61	0.66	3VZ9	163	0.72	0.64	0.72
2IBL	108	0.65	0.66	0.67	3W4Q	826	0.65	0.6	0.66
2IGD	61	0.57	0.56	0.58	3ZBD	213	0.55	0.49	0.55
2IMF	203	0.53	0.58	0.56	3ZIT	157	0.52	0.42	0.5
2IP6	87	0.6	0.66	0.63	3ZRX	241	0.54	0.6	0.58
2IVY	89	0.51	0.45	0.51	3ZSL	165	0.49	0.57	0.53
2J32	244	0.75	0.79	0.79	3ZZP	74	0.38	0.48	0.42
2J9W	203	0.64	0.58	0.64	3ZZY	226	0.65	0.65	0.68
2JKU	38	0.57	0.71	0.66	4A02	169	0.59	0.65	0.62
2JLI	112	0.62	0.68	0.65	4ACJ	182	0.62	0.66	0.64
2JLJ	121	0.71	0.71	0.74	4AE7	189	0.65	0.7	0.68
2MCM	112	0.71	0.77	0.75	4AM1	359	0.54	0.52	0.55
2NLS	36	0.23	0.47	0.29	4ANN	210	0.44	0.43	0.45
2NR7	193	0.78	0.76	0.79	4AVR	189	0.56	0.53	0.56
2NUH	104	0.72	0.56	0.7	4AXY	56	0.59	0.65	0.62
2O6X	309	0.76	0.76	0.78	4B6G	559	0.69	0.68	0.71
2OA2	140	0.54	0.55	0.56	4B9G	292	0.74	0.74	0.76
2OHW	257	0.56	0.46	0.54	4DD5	412	0.61	0.62	0.63
2OKT	377	0.42	0.42	0.43	4DKN	423	0.66	0.64	0.68
2OL9	6	0.94	0.85	0.94	4DND	93	0.62	0.67	0.65
2PKT	93	0.01	−0.04	−0.01	4DPZ	113	0.7	0.74	0.72
2PLT	98	0.52	0.53	0.54	4DQ7	*338*	0.55	0.6	0.57
2PMR	83	0.6	0.63	0.63	4DT4	170	0.67	0.69	0.69
2POF	428	0.62	0.6	0.66	4EK3	313	0.6	0.58	0.61
2PPN	122	0.64	0.54	0.63	4ERY	318	0.57	0.59	0.59
2PSF	608	0.42	0.42	0.43	4ES1	96	0.69	0.69	0.71
2PTH	193	0.69	0.7	0.71	4EUG	225	0.56	0.55	0.58
2Q4N	1208	0.44	0.43	0.45	4F01	459	0.35	0.26	0.33
2Q52	3296	0.55	0.28	0.52	4F3J	143	0.58	0.63	0.62
2QJL	107	0.54	0.57	0.56	4FR9	145	0.6	0.56	0.61
2R16	185	0.44	0.49	0.46	4G14	5	−0.28	0.45	0.04
2R6Q	149	0.63	0.62	0.65	4G2E	155	0.75	0.72	0.76
2RB8	93	0.67	0.7	0.7	4G5X	584	0.71	0.73	0.74
2RE2	249	0.65	0.66	0.68	4G6C	676	0.56	0.54	0.58
2RFR	166	0.61	0.69	0.66	4G7X	216	0.45	0.4	0.45
2V9V	149	0.53	0.52	0.54	4GA2	183	0.61	0.53	0.61
2VE8	515	0.55	0.55	0.58	4GMQ	94	0.76	0.67	0.76
2VH7	94	0.75	0.56	0.73	4GS3	90	0.61	0.56	0.61
2VIM	114	0.44	0.47	0.47	4H4J	278	0.75	0.74	0.77
2VPA	217	0.66	0.75	0.71	4H89	175	0.53	0.58	0.56
2VQ4	106	0.7	0.75	0.72	4HDE	167	0.66	0.72	0.7
2VY8	162	0.77	0.68	0.76	4HJP	308	0.68	0.6	0.67
2VYO	207	0.6	0.63	0.63	4HWM	129	0.54	0.6	0.57
2W1V	551	0.64	0.69	0.66	4IL7	99	0.55	0.55	0.56
2W2A	350	0.59	0.6	0.61	4J11	377	0.58	0.49	0.58
2W6A	139	0.71	0.69	0.72	4J5O	268	0.67	0.68	0.69
2WJ5	110	0.45	0.53	0.48	4J5Q	162	0.72	0.74	0.74
2WUJ	103	0.35	0.54	0.45	4J78	305	0.63	0.6	0.64
2WW7	161	0.36	0.35	0.37	4JG2	202	0.72	0.72	0.73
2WWE	120	0.49	0.55	0.53	4JVU	207	0.7	0.7	0.72
2X1Q	240	0.44	0.5	0.47	4JYP	550	0.59	0.67	0.65
2X25	167	0.5	0.57	0.55	4KEF	145	0.48	0.53	0.51
2X3M	175	0.64	0.65	0.65	5CYT	103	0.39	0.34	0.39
					6RXN	45	0.59	0.6	0.61

**Table 12:** Pearson correlation coefficients of least squares fitting C_*α*_ B-factor prediction of small proteins using 11Å cutoff. Two Bottleneck (B) and Wasserstein (W) metrics using various kernel choices are included.

		B & W			B			W		
PDB ID	*N*	Exp	Lor	Both	Exp	Lor	Both	Exp	Lor	Both
1AIE	31	0.97	0.88	0.99	0.78	0.64	0.90	0.90	0.77	0.96
1AKG	16	0.82	0.66	1.00	0.60	0.53	0.72	0.53	0.56	0.87
1BX7	51	0.86	0.74	0.89	0.79	0.68	0.82	0.81	0.69	0.82
1ETL	12	1.00	1.00	1.00	0.68	0.87	1.00	0.95	0.98	1.00
1ETM	12	1.00	1.00	1.00	0.45	0.74	0.86	0.70	0.83	1.00
1ETN	12	1.00	1.00	1.00	0.96	0.92	0.99	0.70	0.92	1.00
1FF4	65	0.77	0.72	0.80	0.70	0.65	0.75	0.68	0.68	0.76
1GK7	39	0.95	0.94	0.98	0.91	0.93	0.95	0.88	0.92	0.94
1GVD	56	0.75	0.68	0.84	0.67	0.63	0.69	0.61	0.62	0.66
1HJE	13	1.00	1.00	1.00	0.72	0.79	1.00	0.67	0.57	1.00
1KYC	15	0.96	0.99	1.00	0.92	0.93	0.99	0.88	0.88	1.00
1NOT	13	1.00	1.00	1.00	0.82	0.86	1.00	0.86	0.81	1.00
1O06	22	0.98	0.97	1.00	0.96	0.92	0.97	0.97	0.94	0.98
1P9I	29	0.89	0.88	0.98	0.87	0.82	0.92	0.87	0.84	0.89
1PEF	18	0.96	0.97	1.00	0.88	0.94	0.96	0.92	0.94	0.96
1PEN	16	0.96	0.90	1.00	0.60	0.67	0.83	0.47	0.73	0.94
1Q9B	44	0.79	0.76	0.94	0.58	0.59	0.69	0.69	0.57	0.71
1RJU	36	0.81	0.74	0.91	0.75	0.69	0.81	0.62	0.65	0.72
1U06	55	0.50	0.52	0.72	0.37	0.36	0.52	0.46	0.39	0.55
1UOY	64	0.73	0.72	0.83	0.65	0.66	0.69	0.65	0.69	0.73
1USE	47	0.66	0.75	0.91	0.50	0.52	0.72	0.46	0.53	0.64
1VRZ	13	1.00	1.00	1.00	0.92	0.92	1.00	0.77	0.85	1.00
1XY2	8	1.00	1.00	1.00	0.99	0.95	1.00	0.91	0.91	1.00
1YJO	6	1.00	1.00	1.00	1.00	1.00	1.00	1.00	1.00	1.00
1YZM	46	0.87	0.90	0.95	0.82	0.72	0.88	0.86	0.84	0.90
2DSX	52	0.54	0.50	0.78	0.37	0.30	0.56	0.41	0.36	0.55
2JKU	38	0.89	0.75	0.95	0.85	0.65	0.88	0.83	0.60	0.88
2NLS	36	0.75	0.66	0.88	0.61	0.32	0.76	0.49	0.47	0.69
2OL9	6	1.00	1.00	1.00	1.00	1.00	1.00	1.00	1.00	1.00
6RXN	45	0.74	0.63	0.86	0.59	0.48	0.76	0.49	0.49	0.76

**Table 13:** Pearson correlation coefficients of least squares fitting C_*α*_ B-factor prediction of medium proteins using 11Å cutoff. Two Bottleneck (B) and Wasserstein (W) metrics using various kernel choices are included.

		B & W			B			W		
PDB ID	*N*	Exp	Lor	Both	Exp	Lor	Both	Exp	Lor	Both
1ABA	87	0.67	0.67	0.76	0.54	0.62	0.68	0.56	0.63	0.70
1CYO	88	0.71	0.69	0.78	0.66	0.58	0.68	0.65	0.59	0.67
1FK5	93	0.53	0.59	0.71	0.49	0.50	0.58	0.49	0.50	0.55
1GXU	89	0.75	0.78	0.82	0.72	0.61	0.75	0.69	0.72	0.77
1I71	83	0.44	0.66	0.76	0.41	0.46	0.56	0.38	0.58	0.59
1LR7	73	0.61	0.62	0.71	0.57	0.55	0.63	0.46	0.56	0.58
1N7E	95	0.67	0.71	0.80	0.54	0.68	0.72	0.54	0.63	0.73
1NNX	93	0.84	0.84	0.88	0.81	0.79	0.83	0.81	0.81	0.86
1NOA	113	0.63	0.65	0.72	0.60	0.57	0.63	0.53	0.57	0.59
1OPD	85	0.35	0.29	0.57	0.26	0.21	0.36	0.29	0.19	0.36
1QAU	112	0.59	0.61	0.66	0.57	0.55	0.58	0.55	0.57	0.58
1R7J	90	0.88	0.86	0.91	0.83	0.76	0.87	0.81	0.79	0.86
1UHA	82	0.70	0.75	0.82	0.69	0.68	0.74	0.67	0.69	0.73
1ULR	87	0.56	0.53	0.68	0.49	0.50	0.59	0.44	0.50	0.61
1USM	77	0.62	0.61	0.81	0.57	0.53	0.66	0.61	0.58	0.65
1V05	96	0.67	0.66	0.72	0.60	0.61	0.65	0.52	0.61	0.65
1W2L	97	0.72	0.72	0.79	0.60	0.63	0.69	0.56	0.61	0.69
1X3O	80	0.66	0.66	0.72	0.62	0.60	0.65	0.62	0.64	0.67
1Z21	96	0.70	0.73	0.82	0.61	0.63	0.64	0.64	0.69	0.72
1ZVA	75	0.85	0.85	0.94	0.84	0.78	0.92	0.83	0.81	0.86
2BF9	35	0.94	0.73	0.97	0.70	0.65	0.78	0.89	0.71	0.92
2BRF	103	0.74	0.73	0.76	0.74	0.71	0.74	0.72	0.72	0.75
2CE0	109	0.77	0.79	0.86	0.75	0.73	0.80	0.71	0.77	0.79
2E3H	81	0.66	0.71	0.82	0.62	0.69	0.76	0.56	0.69	0.78
2EAQ	89	0.81	0.77	0.86	0.79	0.72	0.81	0.77	0.76	0.82
2EHS	75	0.75	0.73	0.81	0.72	0.71	0.74	0.69	0.71	0.73
2FQ3	85	0.78	0.76	0.82	0.75	0.75	0.79	0.68	0.75	0.78
2IP6	87	0.72	0.66	0.82	0.67	0.58	0.73	0.64	0.64	0.78
2MCM	112	0.80	0.80	0.85	0.78	0.77	0.81	0.75	0.77	0.82
2NUH	104	0.77	0.74	0.85	0.73	0.63	0.81	0.75	0.66	0.80
2PKT	93	0.44	0.39	0.69	0.39	0.35	0.55	0.36	0.36	0.43
2PLT	98	0.66	0.63	0.72	0.57	0.59	0.67	0.52	0.59	0.66
2QJL	107	0.45	0.52	0.63	0.42	0.46	0.50	0.41	0.49	0.51
2RB8	93	0.81	0.78	0.84	0.78	0.75	0.80	0.74	0.76	0.81
3BZQ	99	0.57	0.62	0.69	0.50	0.55	0.61	0.47	0.55	0.59
5CYT	103	0.53	0.52	0.65	0.49	0.46	0.54	0.43	0.48	0.50

**Table 14:** Pearson correlation coefficients of least squares fitting C_*α*_ B-factor prediction of large proteins using 11Å cutoff. Two Bottleneck (B) and Wasserstein (W) metrics using various kernel choices are included.

		B & W			B			W		
PDB ID	*N*	Exp	Lor	Both	Exp	Lor	Both	Exp	Lor	Both
1AHO	66	0.75	0.78	0.88	0.72	0.73	0.79	0.53	0.65	0.75
1ATG	231	0.50	0.50	0.61	0.45	0.47	0.53	0.38	0.48	0.51
1BYI	238	0.50	0.51	0.58	0.41	0.46	0.49	0.44	0.48	0.54
1CCR	109	0.65	0.66	0.71	0.53	0.56	0.65	0.43	0.58	0.63
1E5K	188	0.67	0.68	0.74	0.66	0.67	0.68	0.63	0.67	0.69
1EW4	106	0.58	0.60	0.73	0.52	0.51	0.55	0.55	0.55	0.62
1IFR	113	0.65	0.59	0.73	0.56	0.54	0.65	0.47	0.53	0.62
1NLS	238	0.81	0.78	0.86	0.75	0.65	0.83	0.80	0.72	0.82
1O08	221	0.46	0.48	0.56	0.44	0.42	0.50	0.37	0.45	0.48
1PMY	123	0.71	0.70	0.76	0.62	0.59	0.67	0.68	0.69	0.71
1PZ4	113	0.88	0.82	0.93	0.86	0.74	0.89	0.85	0.76	0.88
1QTO	122	0.59	0.59	0.65	0.48	0.46	0.53	0.55	0.52	0.56
1RRO	108	0.39	0.35	0.56	0.31	0.23	0.45	0.33	0.19	0.45
1UKU	102	0.80	0.81	0.84	0.78	0.80	0.80	0.74	0.80	0.80
1V70	105	0.64	0.65	0.75	0.56	0.60	0.66	0.51	0.58	0.62
1WBE	206	0.53	0.47	0.63	0.43	0.38	0.55	0.36	0.42	0.48
1WHI	122	0.57	0.55	0.63	0.42	0.44	0.57	0.34	0.43	0.55
1WPA	107	0.70	0.69	0.79	0.61	0.52	0.71	0.66	0.56	0.70
2AGK	233	0.65	0.65	0.69	0.61	0.64	0.65	0.55	0.63	0.67
2C71	225	0.45	0.38	0.56	0.29	0.33	0.42	0.23	0.30	0.48
2CG7	110	0.32	0.44	0.63	0.29	0.31	0.36	0.30	0.33	0.41
2CWS	235	0.59	0.55	0.66	0.53	0.52	0.54	0.40	0.52	0.55
2HQK	232	0.80	0.79	0.83	0.70	0.74	0.80	0.68	0.76	0.81
2HYK	237	0.59	0.58	0.63	0.51	0.55	0.59	0.43	0.54	0.60
2I24	113	0.47	0.44	0.69	0.40	0.40	0.48	0.45	0.40	0.49
2IMF	203	0.61	0.65	0.71	0.59	0.56	0.60	0.59	0.59	0.64
2PPN	122	0.57	0.61	0.74	0.51	0.59	0.63	0.44	0.57	0.63
2R16	185	0.50	0.51	0.66	0.46	0.45	0.51	0.45	0.46	0.52
2V9V	149	0.60	0.51	0.66	0.53	0.48	0.56	0.55	0.50	0.62
2VIM	114	0.38	0.33	0.52	0.29	0.28	0.41	0.24	0.31	0.40
2VPA	217	0.73	0.75	0.78	0.72	0.71	0.73	0.68	0.73	0.74
2VYO	207	0.68	0.70	0.77	0.64	0.66	0.72	0.59	0.68	0.70
3SEB	238	0.63	0.66	0.77	0.62	0.61	0.68	0.61	0.62	0.67
3VUB	101	0.65	0.60	0.71	0.60	0.56	0.61	0.61	0.57	0.64

**Table 15:** Pearson correlation coefficients of least squares fitting Cα B-factor prediction of all proteins using 11Å cutoff. Two Bottleneck (B) and Wasserstein (W) metrics using various kernel choices are included.

	B & W			B			W			
PDB ID	*N*	Exp	Lor	Both	Exp	Lor	Both	Exp	Lor	Both
1ABA	87	0.67	0.67	0.76	0.54	0.62	0.68	0.56	0.63	0.70
1AHO	66	0.75	0.78	0.88	0.72	0.73	0.79	0.53	0.65	0.75
1AIE	31	0.97	0.88	0.99	0.78	0.64	0.90	0.90	0.77	0.96
1AKG	16	0.82	0.66	1.00	0.60	0.53	0.72	0.53	0.56	0.87
1ATG	231	0.50	0.50	0.61	0.45	0.47	0.53	0.38	0.48	0.51
1BGF	124	0.75	0.70	0.82	0.64	0.54	0.75	0.68	0.61	0.75
1BX7	51	0.86	0.74	0.89	0.79	0.68	0.82	0.81	0.69	0.82
1BYI	238	0.50	0.51	0.58	0.41	0.46	0.49	0.44	0.48	0.54
1CCR	109	0.65	0.66	0.71	0.53	0.56	0.65	0.43	0.58	0.63
1CYO	88	0.71	0.69	0.78	0.66	0.58	0.68	0.65	0.59	0.67
1DF4	57	0.93	0.92	0.97	0.92	0.89	0.95	0.88	0.91	0.94
1E5K	188	0.67	0.68	0.74	0.66	0.67	0.68	0.63	0.67	0.69
1ES5	260	0.58	0.57	0.65	0.51	0.55	0.58	0.44	0.56	0.60
1ETL	12	1.00	1.00	1.00	0.68	0.87	1.00	0.95	0.98	1.00
1ETM	12	1.00	1.00	1.00	0.45	0.74	0.86	0.70	0.83	1.00
1ETN	12	1.00	1.00	1.00	0.96	0.92	0.99	0.70	0.92	1.00
1EW4	106	0.58	0.60	0.73	0.52	0.51	0.55	0.55	0.55	0.62
1F8R	1932	0.61	0.63	0.70	0.59	0.62	0.63	0.50	0.62	0.65
1FF4	65	0.77	0.72	0.80	0.70	0.65	0.75	0.68	0.68	0.76
1FK5	93	0.53	0.59	0.71	0.49	0.50	0.58	0.49	0.50	0.55
1GCO	1044	0.63	0.64	0.66	0.59	0.63	0.63	0.53	0.63	0.65
1GK7	39	0.95	0.94	0.98	0.91	0.93	0.95	0.88	0.92	0.94
1GVD	56	0.75	0.68	0.84	0.67	0.63	0.69	0.61	0.62	0.66
1GXU	89	0.75	0.78	0.82	0.72	0.61	0.75	0.69	0.72	0.77
1H6V	2927	0.29	0.31	0.33	0.28	0.29	0.30	0.23	0.29	0.30
1HJE	13	1.00	1.00	1.00	0.72	0.79	1.00	0.67	0.57	1.00
1I71	83	0.44	0.66	0.76	0.41	0.46	0.56	0.38	0.58	0.59
1IDP	441	0.48	0.47	0.55	0.43	0.45	0.47	0.39	0.46	0.48
1IFR	113	0.65	0.59	0.73	0.56	0.54	0.65	0.47	0.53	0.62
1K8U	87	0.72	0.74	0.85	0.67	0.64	0.71	0.65	0.67	0.75
1KMM	1499	0.57	0.54	0.59	0.49	0.53	0.54	0.36	0.53	0.57
1KNG	144	0.52	0.51	0.61	0.43	0.47	0.51	0.43	0.50	0.53
1KR4	107	0.57	0.48	0.60	0.39	0.47	0.53	0.45	0.45	0.54
1KYC	15	0.96	0.99	1.00	0.92	0.93	0.99	0.88	0.88	1.00
1LR7	73	0.61	0.62	0.71	0.57	0.55	0.63	0.46	0.56	0.58
1MF7	194	0.56	0.59	0.67	0.55	0.57	0.59	0.50	0.58	0.59
1N7E	95	0.67	0.71	0.80	0.54	0.68	0.72	0.54	0.63	0.73
1NKD	59	0.73	0.69	0.89	0.56	0.58	0.63	0.55	0.65	0.75
1NLS	238	0.81	0.78	0.86	0.75	0.65	0.83	0.80	0.72	0.82
1NNX	93	0.84	0.84	0.88	0.81	0.79	0.83	0.81	0.81	0.86
1NOA	113	0.63	0.65	0.72	0.60	0.57	0.63	0.53	0.57	0.59
1NOT	13	1.00	1.00	1.00	0.82	0.86	1.00	0.86	0.81	1.00
1O06	22	0.98	0.97	1.00	0.96	0.92	0.97	0.97	0.94	0.98
1O08	221	0.46	0.48	0.56	0.44	0.42	0.50	0.37	0.45	0.48
1OB4	5	1.00	1.00	1.00	1.00	1.00	1.00	1.00	1.00	1.00
1OB7	5	1.00	1.00	1.00	1.00	1.00	1.00	1.00	1.00	1.00
1OPD	85	0.35	0.29	0.57	0.25	0.21	0.36	0.29	0.19	0.36
1P9I	29	0.89	0.88	0.98	0.87	0.82	0.92	0.87	0.84	0.89
1PEF	18	0.96	0.97	1.00	0.88	0.94	0.96	0.92	0.94	0.96
1PEN	16	0.96	0.90	1.00	0.60	0.67	0.83	0.47	0.73	0.94
1PMY	123	0.71	0.70	0.76	0.62	0.59	0.67	0.68	0.69	0.71
1PZ4	113	0.88	0.82	0.93	0.86	0.74	0.89	0.85	0.76	0.88
1Q9B	44	0.79	0.76	0.94	0.58	0.59	0.69	0.69	0.57	0.71
1QAU	112	0.59	0.61	0.66	0.57	0.55	0.58	0.55	0.57	0.58
1QKI	3912	0.38	0.42	0.45	0.34	0.38	0.41	0.32	0.38	0.40
1QTO	122	0.59	0.59	0.65	0.48	0.46	0.53	0.55	0.52	0.56
1R29	122	0.71	0.56	0.76	0.55	0.35	0.69	0.69	0.43	0.72
1R7J	90	0.88	0.86	0.91	0.83	0.76	0.87	0.81	0.79	0.86
1RJU	36	0.81	0.74	0.91	0.75	0.69	0.81	0.62	0.65	0.72
1RRO	108	0.39	0.35	0.56	0.31	0.23	0.45	0.33	0.19	0.45
1SAU	123	0.76	0.75	0.81	0.70	0.73	0.75	0.68	0.74	0.76
1TGR	111	0.77	0.76	0.83	0.72	0.70	0.74	0.74	0.73	0.75
1TZV	157	0.76	0.78	0.83	0.73	0.71	0.77	0.69	0.70	0.74
1U06	55	0.50	0.52	0.72	0.37	0.36	0.52	0.46	0.39	0.55
1U7I	259	0.71	0.71	0.73	0.62	0.68	0.70	0.53	0.67	0.71
1U9C	220	0.66	0.65	0.74	0.61	0.57	0.64	0.61	0.60	0.67
1UHA	82	0.70	0.75	0.82	0.69	0.68	0.74	0.67	0.69	0.73
1UKU	102	0.80	0.81	0.84	0.78	0.80	0.80	0.74	0.80	0.80
1ULR	87	0.56	0.53	0.68	0.49	0.50	0.59	0.44	0.50	0.61
1UOY	64	0.73	0.72	0.83	0.65	0.66	0.69	0.65	0.69	0.73
1USE	47	0.66	0.75	0.91	0.50	0.52	0.72	0.46	0.53	0.64
1USM	77	0.62	0.61	0.81	0.57	0.53	0.66	0.61	0.58	0.65
1UTG	70	0.57	0.53	0.68	0.51	0.49	0.60	0.49	0.49	0.56
1V05	96	0.67	0.66	0.72	0.60	0.61	0.65	0.52	0.61	0.65
1V70	105	0.64	0.65	0.75	0.56	0.60	0.66	0.51	0.58	0.62
1VRZ	13	1.00	1.00	1.00	0.92	0.92	1.00	0.77	0.85	1.00
1W2L	97	0.72	0.72	0.79	0.60	0.63	0.69	0.56	0.61	0.69
1WBE	206	0.53	0.47	0.63	0.43	0.38	0.55	0.36	0.42	0.48
1WHI	122	0.57	0.55	0.63	0.42	0.44	0.57	0.34	0.43	0.55
1WLY	322	0.62	0.64	0.67	0.59	0.62	0.63	0.54	0.62	0.64
1WPA	107	0.70	0.69	0.79	0.61	0.52	0.71	0.66	0.56	0.70
1X3O	80	0.66	0.66	0.72	0.62	0.60	0.65	0.62	0.64	0.67
1XY1	16	0.97	0.96	1.00	0.73	0.66	0.87	0.81	0.89	0.99
1XY2	8	1.00	1.00	1.00	0.99	0.95	1.00	0.91	0.91	1.00
1Y6X	86	0.56	0.53	0.62	0.50	0.49	0.59	0.50	0.52	0.56
1YJO	6	1.00	1.00	1.00	1.00	1.00	1.00	1.00	1.00	1.00
1YZM	46	0.87	0.90	0.95	0.82	0.72	0.88	0.86	0.84	0.90
1Z21	96	0.70	0.73	0.82	0.61	0.63	0.64	0.64	0.69	0.72
1ZCE	139	0.84	0.83	0.88	0.83	0.77	0.85	0.81	0.78	0.82
1ZVA	75	0.85	0.85	0.94	0.84	0.78	0.92	0.83	0.81	0.86
2A50	469	0.64	0.63	0.70	0.54	0.60	0.67	0.41	0.58	0.67
2AGK	233	0.65	0.65	0.69	0.61	0.64	0.65	0.55	0.63	0.67
2AH1	939	0.45	0.47	0.49	0.42	0.45	0.46	0.33	0.46	0.48
2B0A	191	0.59	0.60	0.69	0.50	0.58	0.62	0.48	0.59	0.63
2BCM	415	0.46	0.41	0.50	0.39	0.39	0.40	0.35	0.39	0.45
2BF9	35	0.94	0.73	0.97	0.70	0.65	0.78	0.89	0.71	0.92
2BRF	103	0.74	0.73	0.76	0.74	0.71	0.74	0.72	0.72	0.75
2C71	225	0.45	0.38	0.56	0.29	0.33	0.42	0.23	0.30	0.48
2CE0	109	0.77	0.79	0.86	0.75	0.73	0.80	0.71	0.77	0.79
2CG7	110	0.32	0.44	0.63	0.29	0.31	0.36	0.30	0.33	0.41
2COV	534	0.66	0.64	0.70	0.63	0.64	0.67	0.57	0.64	0.67
2CWS	235	0.59	0.55	0.66	0.53	0.52	0.54	0.40	0.52	0.55
2D5W	1214	0.52	0.52	0.54	0.49	0.52	0.52	0.41	0.52	0.53
2DKO	253	0.75	0.72	0.79	0.72	0.69	0.75	0.68	0.69	0.72
2DPL	565	0.35	0.36	0.41	0.30	0.32	0.35	0.24	0.33	0.37
2DSX	52	0.54	0.50	0.78	0.37	0.30	0.56	0.41	0.36	0.55
2E10	439	0.60	0.59	0.65	0.51	0.58	0.61	0.43	0.57	0.62
2E3H	81	0.66	0.71	0.82	0.62	0.69	0.76	0.56	0.69	0.78
2EAQ	89	0.81	0.77	0.86	0.78	0.72	0.81	0.77	0.76	0.82
2EHP	246	0.63	0.65	0.71	0.58	0.62	0.65	0.52	0.62	0.64
2EHS	75	0.75	0.73	0.81	0.72	0.71	0.74	0.69	0.71	0.73
2ERW	53	0.62	0.41	0.84	0.33	0.26	0.60	0.31	0.28	0.49
2ETX	390	0.54	0.54	0.57	0.52	0.53	0.56	0.47	0.51	0.54
2FB6	129	0.71	0.66	0.76	0.67	0.63	0.69	0.65	0.63	0.74
2FG1	176	0.55	0.56	0.62	0.54	0.52	0.58	0.52	0.54	0.57
2FN9	560	0.51	0.49	0.62	0.44	0.47	0.55	0.41	0.46	0.55
2FQ3	85	0.78	0.76	0.82	0.75	0.75	0.79	0.68	0.75	0.78
2G69	99	0.59	0.65	0.76	0.42	0.50	0.66	0.47	0.45	0.60
2G7O	68	0.89	0.91	0.95	0.85	0.79	0.88	0.76	0.82	0.87
2G7S	206	0.63	0.60	0.66	0.59	0.58	0.63	0.54	0.59	0.63
2GKG	150	0.77	0.71	0.83	0.74	0.65	0.78	0.76	0.67	0.78
2GOM	121	0.47	0.52	0.64	0.42	0.42	0.45	0.44	0.47	0.53
2GXG	140	0.74	0.72	0.79	0.71	0.68	0.72	0.69	0.68	0.73
2GZQ	203	0.45	0.40	0.60	0.38	0.34	0.48	0.24	0.29	0.31
2HQK	232	0.80	0.79	0.83	0.70	0.74	0.80	0.68	0.76	0.81
2HYK	237	0.59	0.58	0.63	0.51	0.55	0.59	0.43	0.54	0.60
2I24	113	0.47	0.44	0.69	0.40	0.40	0.48	0.45	0.40	0.49
2I49	399	0.54	0.53	0.62	0.43	0.51	0.56	0.41	0.49	0.58
2IBL	108	0.69	0.71	0.75	0.66	0.67	0.70	0.65	0.68	0.71
2IGD	61	0.67	0.72	0.84	0.61	0.64	0.74	0.61	0.66	0.74
2IMF	203	0.61	0.65	0.71	0.59	0.56	0.60	0.59	0.59	0.64
2IP6	87	0.72	0.66	0.82	0.66	0.58	0.73	0.64	0.64	0.78
2IVY	89	0.43	0.53	0.69	0.35	0.45	0.48	0.34	0.42	0.57
2J32	244	0.77	0.72	0.85	0.73	0.68	0.77	0.73	0.68	0.77
2J9W	203	0.59	0.60	0.70	0.55	0.59	0.64	0.51	0.59	0.62
2JKU	38	0.89	0.75	0.95	0.85	0.65	0.88	0.83	0.60	0.88
2JLI	112	0.87	0.81	0.90	0.82	0.70	0.85	0.85	0.78	0.86
2JLJ	121	0.78	0.75	0.80	0.71	0.65	0.74	0.74	0.71	0.76
2MCM	112	0.80	0.80	0.85	0.78	0.77	0.81	0.75	0.77	0.82
2NLS	36	0.75	0.66	0.88	0.61	0.32	0.76	0.49	0.47	0.69
2NR7	193	0.75	0.75	0.79	0.74	0.72	0.76	0.71	0.73	0.77
2NUH	104	0.77	0.74	0.85	0.73	0.63	0.81	0.75	0.66	0.80
2O6X	309	0.74	0.75	0.78	0.70	0.73	0.75	0.65	0.73	0.75
2OA2	140	0.63	0.64	0.70	0.55	0.49	0.60	0.60	0.63	0.67
2OHW	257	0.35	0.39	0.48	0.29	0.32	0.35	0.27	0.34	0.38
2OKT	377	0.43	0.37	0.49	0.31	0.36	0.40	0.22	0.33	0.46
2OL9	6	1.00	1.00	1.00	1.00	1.00	1.00	1.00	1.00	1.00
2OLX	4	1.00	1.00	1.00	1.00	1.00	1.00	1.00	1.00	1.00
2PKT	93	0.44	0.39	0.69	0.40	0.35	0.55	0.36	0.36	0.43
2PLT	98	0.66	0.63	0.72	0.57	0.59	0.67	0.52	0.59	0.66
2PMR	83	0.69	0.68	0.80	0.59	0.62	0.68	0.65	0.65	0.69
2POF	428	0.62	0.56	0.66	0.48	0.55	0.60	0.44	0.54	0.63
2PPN	122	0.57	0.61	0.74	0.51	0.59	0.63	0.44	0.57	0.63
2PSF	608	0.43	0.45	0.53	0.41	0.44	0.45	0.37	0.42	0.44
2PTH	193	0.71	0.71	0.77	0.65	0.70	0.73	0.61	0.69	0.72
2Q4N	1208	0.65	0.62	0.68	0.58	0.55	0.59	0.55	0.57	0.61
2Q52	3296	0.65	0.66	0.70	0.62	0.56	0.64	0.63	0.57	0.65
2QJL	107	0.45	0.52	0.63	0.42	0.46	0.50	0.41	0.49	0.51
2R16	185	0.50	0.51	0.66	0.46	0.45	0.51	0.45	0.46	0.52
2R6Q	149	0.71	0.72	0.76	0.66	0.68	0.70	0.62	0.65	0.67
2RB8	93	0.81	0.78	0.84	0.78	0.75	0.80	0.74	0.76	0.81
2RE2	249	0.64	0.65	0.70	0.57	0.59	0.61	0.59	0.60	0.63
2RFR	166	0.73	0.66	0.80	0.68	0.57	0.74	0.72	0.59	0.74
2V9V	149	0.60	0.51	0.66	0.53	0.48	0.56	0.55	0.50	0.62
2VE8	515	0.46	0.48	0.55	0.42	0.41	0.44	0.40	0.43	0.47
2VH7	94	0.59	0.54	0.68	0.52	0.49	0.63	0.42	0.49	0.54
2VIM	114	0.38	0.33	0.52	0.29	0.28	0.41	0.24	0.31	0.40
2VPA	217	0.73	0.75	0.78	0.72	0.71	0.73	0.68	0.73	0.74
2VQ4	106	0.56	0.54	0.64	0.43	0.49	0.56	0.35	0.46	0.58
2VY8	162	0.47	0.46	0.58	0.38	0.42	0.46	0.38	0.42	0.49
2VYO	207	0.68	0.70	0.77	0.64	0.66	0.72	0.59	0.68	0.70
2W1V	551	0.69	0.67	0.77	0.63	0.63	0.70	0.56	0.64	0.68
2W2A	350	0.60	0.59	0.65	0.57	0.56	0.59	0.54	0.57	0.60
2W6A	139	0.59	0.59	0.64	0.51	0.52	0.54	0.52	0.56	0.60
2WJ5	110	0.63	0.55	0.79	0.59	0.52	0.68	0.59	0.53	0.64
2WUJ	103	0.69	0.68	0.79	0.62	0.52	0.65	0.67	0.59	0.71
2WW7	161	0.44	0.48	0.60	0.40	0.42	0.50	0.33	0.43	0.49
2WWE	120	0.71	0.71	0.83	0.62	0.62	0.75	0.61	0.58	0.73
2X1Q	240	0.48	0.44	0.54	0.38	0.39	0.46	0.34	0.37	0.47
2X25	167	0.62	0.61	0.73	0.56	0.57	0.64	0.57	0.57	0.64
2X3M	175	0.61	0.61	0.69	0.60	0.55	0.64	0.57	0.57	0.60
2X5Y	185	0.67	0.63	0.71	0.60	0.59	0.64	0.53	0.58	0.69
2X9Z	266	0.50	0.42	0.54	0.37	0.38	0.42	0.38	0.39	0.51
2XHF	310	0.62	0.62	0.67	0.58	0.56	0.60	0.55	0.62	0.63
2Y0T	111	0.69	0.68	0.83	0.60	0.61	0.68	0.56	0.64	0.70
2Y72	183	0.71	0.71	0.78	0.69	0.69	0.72	0.66	0.70	0.71
2Y7L	323	0.68	0.70	0.72	0.66	0.68	0.69	0.58	0.69	0.69
2Y9F	149	0.75	0.72	0.78	0.65	0.69	0.71	0.58	0.70	0.74
2YLB	418	0.55	0.52	0.63	0.46	0.49	0.52	0.34	0.49	0.59
2YNY	326	0.63	0.67	0.75	0.60	0.62	0.63	0.56	0.63	0.66
2ZCM	348	0.42	0.39	0.49	0.34	0.35	0.40	0.24	0.32	0.43
2ZU1	360	0.61	0.61	0.68	0.53	0.58	0.63	0.45	0.58	0.63
3A0M	146	0.74	0.76	0.84	0.68	0.70	0.72	0.61	0.73	0.78
3A7L	128	0.69	0.61	0.78	0.52	0.45	0.59	0.62	0.54	0.67
3AMC	614	0.54	0.53	0.64	0.47	0.50	0.54	0.37	0.51	0.57
3AUB	124	0.36	0.41	0.53	0.31	0.26	0.41	0.32	0.32	0.37
3B5O	249	0.55	0.58	0.66	0.52	0.56	0.63	0.46	0.55	0.57
3BA1	312	0.67	0.66	0.72	0.64	0.65	0.68	0.60	0.65	0.70
3BED	262	0.61	0.55	0.67	0.53	0.53	0.56	0.44	0.53	0.61
3BQX	136	0.52	0.50	0.54	0.47	0.48	0.51	0.41	0.46	0.51
3BZQ	99	0.57	0.62	0.69	0.50	0.55	0.61	0.47	0.55	0.59
3BZZ	103	0.60	0.63	0.68	0.51	0.58	0.61	0.45	0.50	0.59
3DRF	567	0.32	0.32	0.38	0.27	0.29	0.33	0.22	0.30	0.34
3DWV	359	0.67	0.63	0.69	0.62	0.62	0.66	0.54	0.62	0.65
3E5T	268	0.55	0.52	0.60	0.51	0.51	0.56	0.38	0.50	0.55
3E7R	40	0.81	0.86	0.96	0.78	0.77	0.81	0.73	0.82	0.88
3EUR	150	0.49	0.46	0.53	0.39	0.43	0.47	0.31	0.42	0.47
3F2Z	148	0.76	0.78	0.84	0.75	0.76	0.78	0.69	0.77	0.78
3F7E	261	0.66	0.65	0.71	0.61	0.64	0.65	0.47	0.63	0.69
3FCN	185	0.60	0.65	0.75	0.56	0.59	0.64	0.54	0.59	0.67
3FE7	89	0.69	0.65	0.76	0.58	0.60	0.67	0.54	0.63	0.70
3FKE	250	0.47	0.42	0.52	0.40	0.36	0.49	0.34	0.36	0.45
3FMY	75	0.71	0.69	0.79	0.66	0.64	0.70	0.66	0.66	0.71
3FOD	48	0.48	0.47	0.82	0.42	0.33	0.55	0.38	0.35	0.48
3FSO	238	0.82	0.82	0.85	0.77	0.74	0.77	0.77	0.81	0.82
3FTD	257	0.60	0.57	0.67	0.49	0.52	0.59	0.41	0.52	0.60
3G1S	418	0.44	0.51	0.68	0.41	0.45	0.51	0.38	0.45	0.49
3GBW	170	0.77	0.78	0.84	0.64	0.74	0.79	0.51	0.71	0.81
3GHJ	129	0.71	0.71	0.81	0.65	0.67	0.72	0.65	0.68	0.72
3HFO	216	0.75	0.72	0.82	0.70	0.63	0.75	0.65	0.69	0.74
3HHP	1314	0.61	0.62	0.68	0.57	0.59	0.62	0.52	0.59	0.63
3HNY	170	0.59	0.56	0.64	0.47	0.52	0.57	0.42	0.49	0.56
3HP4	201	0.60	0.61	0.72	0.57	0.54	0.64	0.43	0.56	0.62
3HWU	155	0.60	0.69	0.81	0.57	0.61	0.63	0.50	0.61	0.68
3HYD	8	1.00	1.00	1.00	1.00	1.00	1.00	1.00	1.00	1.00
3HZ8	200	0.58	0.59	0.66	0.55	0.53	0.56	0.52	0.54	0.58
3I2V	127	0.57	0.58	0.66	0.51	0.53	0.61	0.40	0.48	0.53
3I2Z	140	0.58	0.59	0.65	0.52	0.54	0.56	0.56	0.57	0.61
3I4O	154	0.63	0.64	0.73	0.58	0.59	0.60	0.56	0.63	0.66
3I7M	145	0.58	0.62	0.71	0.53	0.55	0.58	0.49	0.58	0.64
3IHS	173	0.62	0.67	0.74	0.58	0.54	0.60	0.58	0.60	0.62
3IVV	168	0.80	0.80	0.89	0.75	0.76	0.83	0.68	0.74	0.79
3K6Y	227	0.53	0.53	0.60	0.48	0.49	0.52	0.42	0.50	0.55
3KBE	166	0.62	0.61	0.65	0.57	0.60	0.62	0.52	0.60	0.61
3KGK	190	0.79	0.80	0.84	0.77	0.79	0.81	0.68	0.79	0.80
3KZD	94	0.79	0.72	0.83	0.55	0.68	0.77	0.47	0.66	0.78
3L41	219	0.61	0.62	0.71	0.59	0.60	0.66	0.57	0.59	0.67
3LAA	176	0.70	0.66	0.80	0.68	0.56	0.76	0.69	0.60	0.77
3LAX	118	0.81	0.81	0.86	0.80	0.76	0.83	0.77	0.78	0.82
3LG3	846	0.40	0.38	0.41	0.36	0.37	0.40	0.32	0.37	0.41
3LJI	270	0.53	0.53	0.62	0.47	0.52	0.58	0.45	0.52	0.56
3M3P	244	0.47	0.44	0.69	0.40	0.40	0.58	0.25	0.35	0.48
3M8J	178	0.74	0.72	0.75	0.69	0.69	0.73	0.67	0.70	0.73
3M9J	250	0.57	0.56	0.59	0.53	0.54	0.56	0.39	0.53	0.56
3M9Q	190	0.53	0.52	0.59	0.50	0.51	0.53	0.46	0.50	0.51
3MAB	180	0.57	0.56	0.62	0.52	0.47	0.55	0.56	0.51	0.56
3MD4	13	1.00	1.00	1.00	0.91	0.94	1.00	0.93	0.99	1.00
3MD5	14	1.00	1.00	1.00	0.98	0.93	1.00	0.94	0.92	1.00
3MEA	170	0.58	0.58	0.68	0.57	0.57	0.64	0.48	0.57	0.59
3MGN	277	0.33	0.32	0.47	0.26	0.28	0.30	0.16	0.29	0.39
3MRE	446	0.40	0.38	0.45	0.32	0.36	0.40	0.24	0.35	0.41
3N11	325	0.43	0.45	0.51	0.42	0.44	0.45	0.38	0.44	0.45
3NE0	208	0.77	0.79	0.84	0.75	0.70	0.77	0.70	0.76	0.82
3NGG	97	0.80	0.81	0.85	0.72	0.74	0.78	0.74	0.76	0.80
3NPV	500	0.44	0.44	0.50	0.40	0.42	0.44	0.36	0.43	0.47
3NVG	6	1.00	1.00	1.00	1.00	1.00	1.00	1.00	1.00	1.00
3NZL	70	0.68	0.61	0.84	0.53	0.49	0.66	0.59	0.55	0.67
3O0P	197	0.62	0.64	0.71	0.59	0.62	0.64	0.53	0.62	0.64
3O5P	147	0.64	0.60	0.71	0.55	0.57	0.60	0.53	0.56	0.64
3OBQ	150	0.59	0.59	0.66	0.46	0.49	0.58	0.53	0.56	0.58
3OQY	236	0.71	0.66	0.73	0.63	0.64	0.70	0.60	0.64	0.72
3P6J	145	0.75	0.73	0.81	0.69	0.71	0.73	0.61	0.71	0.75
3PD7	216	0.65	0.66	0.72	0.62	0.60	0.65	0.60	0.61	0.65
3PES	166	0.70	0.72	0.79	0.58	0.63	0.70	0.52	0.60	0.66
3PID	387	0.50	0.49	0.56	0.44	0.48	0.53	0.37	0.46	0.51
3PIW	161	0.66	0.67	0.78	0.60	0.63	0.70	0.56	0.63	0.72
3PKV	229	0.50	0.52	0.63	0.43	0.48	0.53	0.35	0.50	0.57
3PSM	94	0.83	0.78	0.88	0.79	0.77	0.83	0.68	0.76	0.79
3PTL	289	0.50	0.50	0.53	0.49	0.49	0.50	0.43	0.49	0.50
3PVE	363	0.45	0.45	0.59	0.37	0.39	0.44	0.41	0.42	0.45
3PZ9	357	0.51	0.45	0.57	0.36	0.38	0.42	0.34	0.39	0.50
3PZZ	12	1.00	1.00	1.00	0.95	0.90	1.00	0.94	0.80	1.00
3Q2X	6	1.00	1.00	1.00	1.00	1.00	1.00	1.00	1.00	1.00
3Q6L	131	0.39	0.44	0.56	0.33	0.31	0.37	0.34	0.37	0.42
3QDS	284	0.63	0.62	0.69	0.59	0.59	0.65	0.51	0.59	0.64
3QPA	212	0.68	0.66	0.78	0.45	0.45	0.47	0.59	0.59	0.65
3R6D	222	0.65	0.66	0.73	0.62	0.63	0.65	0.53	0.64	0.69
3R87	148	0.48	0.47	0.55	0.41	0.44	0.48	0.40	0.45	0.47
3RQ9	165	0.51	0.47	0.61	0.41	0.44	0.52	0.39	0.45	0.56
3RY0	128	0.44	0.45	0.54	0.40	0.40	0.47	0.41	0.42	0.47
3RZY	151	0.65	0.65	0.84	0.59	0.54	0.65	0.57	0.51	0.59
3S0A	132	0.39	0.43	0.52	0.33	0.34	0.38	0.32	0.31	0.37
3SD2	100	0.65	0.67	0.77	0.64	0.63	0.69	0.56	0.63	0.67
3SEB	238	0.63	0.66	0.77	0.62	0.61	0.68	0.61	0.62	0.67
3SED	126	0.39	0.45	0.55	0.28	0.29	0.38	0.33	0.33	0.40
3SO6	157	0.67	0.71	0.78	0.63	0.69	0.73	0.55	0.64	0.70
3SR3	657	0.45	0.44	0.48	0.43	0.41	0.45	0.39	0.43	0.44
3SUK	254	0.53	0.54	0.64	0.46	0.48	0.54	0.47	0.49	0.57
3SZH	753	0.53	0.53	0.57	0.51	0.51	0.52	0.45	0.52	0.53
3T0H	209	0.76	0.73	0.78	0.72	0.69	0.74	0.68	0.71	0.76
3T3K	122	0.66	0.66	0.72	0.55	0.62	0.68	0.48	0.60	0.68
3T47	145	0.54	0.54	0.78	0.45	0.45	0.62	0.43	0.47	0.54
3TDN	359	0.47	0.43	0.53	0.43	0.42	0.44	0.38	0.43	0.49
3TOW	155	0.66	0.65	0.74	0.58	0.61	0.66	0.53	0.60	0.65
3TUA	226	0.57	0.55	0.63	0.52	0.50	0.55	0.45	0.52	0.54
3TYS	78	0.78	0.58	0.86	0.67	0.48	0.73	0.70	0.46	0.75
3U6G	276	0.44	0.39	0.54	0.39	0.37	0.45	0.27	0.35	0.48
3U97	85	0.78	0.78	0.84	0.77	0.73	0.80	0.77	0.76	0.80
3UCI	72	0.67	0.64	0.72	0.48	0.53	0.57	0.55	0.56	0.63
3UR8	637	0.52	0.53	0.60	0.49	0.51	0.55	0.45	0.52	0.53
3US6	159	0.60	0.56	0.67	0.55	0.49	0.62	0.53	0.46	0.59
3V1A	59	0.74	0.57	0.95	0.51	0.53	0.77	0.39	0.46	0.68
3V75	294	0.50	0.49	0.57	0.48	0.46	0.53	0.47	0.47	0.53
3VN0	193	0.87	0.88	0.90	0.86	0.87	0.88	0.79	0.88	0.89
3VOR	219	0.64	0.58	0.70	0.56	0.52	0.63	0.53	0.55	0.63
3VUB	101	0.65	0.60	0.71	0.60	0.56	0.61	0.61	0.57	0.64
3VVV	112	0.64	0.64	0.79	0.55	0.48	0.65	0.57	0.49	0.58
3VZ9	163	0.65	0.64	0.70	0.60	0.55	0.63	0.60	0.60	0.67
3W4Q	826	0.61	0.60	0.68	0.56	0.59	0.61	0.47	0.60	0.64
3ZBD	213	0.36	0.47	0.74	0.24	0.28	0.34	0.25	0.31	0.36
3ZIT	157	0.51	0.47	0.59	0.36	0.39	0.47	0.47	0.41	0.52
3ZRX	241	0.56	0.56	0.63	0.49	0.52	0.53	0.46	0.52	0.56
3ZSL	165	0.39	0.39	0.54	0.28	0.22	0.40	0.31	0.24	0.37
3ZZP	74	0.40	0.30	0.47	0.19	0.27	0.31	0.12	0.22	0.40
3ZZY	226	0.65	0.67	0.69	0.63	0.63	0.64	0.59	0.63	0.64
4A02	169	0.61	0.56	0.66	0.49	0.52	0.57	0.31	0.51	0.60
4ACJ	182	0.55	0.59	0.75	0.55	0.58	0.61	0.51	0.59	0.60
4AE7	189	0.69	0.67	0.74	0.63	0.61	0.65	0.63	0.65	0.69
4AM1	359	0.57	0.54	0.59	0.53	0.52	0.53	0.46	0.53	0.55
4ANN	210	0.50	0.48	0.57	0.42	0.43	0.48	0.36	0.42	0.47
4AVR	189	0.57	0.57	0.70	0.53	0.51	0.59	0.49	0.53	0.57
4AXY	56	0.55	0.60	0.76	0.47	0.48	0.63	0.47	0.50	0.62
4B6G	559	0.70	0.71	0.75	0.67	0.69	0.72	0.60	0.69	0.73
4B9G	292	0.81	0.82	0.85	0.78	0.80	0.81	0.71	0.82	0.83
4DD5	412	0.60	0.63	0.71	0.57	0.59	0.63	0.51	0.61	0.66
4DKN	423	0.59	0.58	0.63	0.52	0.54	0.56	0.42	0.55	0.61
4DND	93	0.75	0.66	0.82	0.67	0.64	0.75	0.61	0.64	0.74
4DPZ	113	0.68	0.70	0.79	0.65	0.64	0.67	0.62	0.64	0.69
4DQ7	338	0.45	0.46	0.51	0.37	0.44	0.49	0.29	0.40	0.46
4DT4	170	0.76	0.74	0.78	0.70	0.68	0.72	0.70	0.70	0.73
4EK3	313	0.58	0.63	0.65	0.55	0.56	0.58	0.53	0.59	0.60
4ERY	318	0.61	0.60	0.67	0.59	0.59	0.64	0.52	0.59	0.65
4ES1	96	0.76	0.77	0.86	0.69	0.73	0.78	0.57	0.74	0.83
4EUG	225	0.61	0.61	0.67	0.54	0.60	0.62	0.51	0.58	0.62
4F01	459	0.38	0.37	0.47	0.32	0.34	0.37	0.22	0.34	0.39
4F3J	143	0.57	0.63	0.66	0.52	0.59	0.61	0.47	0.58	0.60
4FR9	145	0.65	0.62	0.78	0.63	0.58	0.70	0.58	0.57	0.64
4G14	5	1.00	1.00	1.00	1.00	1.00	1.00	1.00	1.00	1.00
4G2E	155	0.75	0.64	0.85	0.59	0.61	0.74	0.68	0.61	0.80
4G5X	584	0.71	0.69	0.80	0.69	0.64	0.74	0.64	0.67	0.72
4G6C	676	0.43	0.44	0.50	0.40	0.44	0.46	0.24	0.43	0.45
4G7X	216	0.53	0.47	0.61	0.41	0.31	0.47	0.51	0.37	0.53
4GA2	183	0.55	0.56	0.70	0.52	0.53	0.57	0.49	0.53	0.60
4GMQ	94	0.73	0.77	0.84	0.68	0.66	0.72	0.67	0.63	0.72
4GS3	90	0.65	0.68	0.74	0.60	0.64	0.68	0.51	0.66	0.70
4H4J	278	0.67	0.67	0.82	0.63	0.64	0.75	0.57	0.66	0.69
4H89	175	0.39	0.50	0.67	0.33	0.37	0.39	0.35	0.40	0.42
4HDE	167	0.63	0.55	0.75	0.59	0.52	0.69	0.59	0.51	0.67
4HJP	308	0.62	0.61	0.65	0.57	0.55	0.59	0.58	0.58	0.62
4HWM	129	0.69	0.66	0.71	0.66	0.60	0.68	0.68	0.63	0.70
4IL7	99	0.63	0.63	0.65	0.60	0.59	0.62	0.57	0.61	0.62
4J11	377	0.66	0.63	0.68	0.62	0.61	0.63	0.63	0.61	0.66
4J5O	268	0.77	0.76	0.82	0.71	0.62	0.77	0.75	0.66	0.77
4J5Q	162	0.65	0.63	0.75	0.57	0.56	0.66	0.59	0.57	0.64
4J78	305	0.48	0.48	0.56	0.43	0.44	0.50	0.38	0.47	0.53
4JG2	202	0.63	0.63	0.74	0.61	0.61	0.64	0.58	0.60	0.63
4JVU	207	0.67	0.64	0.75	0.57	0.58	0.66	0.59	0.60	0.67
4JYP	550	0.59	0.60	0.69	0.52	0.57	0.61	0.38	0.58	0.61
4KEF	145	0.52	0.49	0.65	0.40	0.42	0.49	0.27	0.45	0.56
5CYT	103	0.53	0.52	0.65	0.49	0.46	0.54	0.43	0.48	0.50
6RXN	45	0.74	0.63	0.86	0.59	0.48	0.76	0.49	0.49	0.76
